# Understanding variable disease severity in X-linked retinoschisis: Does RS1 secretory mechanism determine disease severity?

**DOI:** 10.1371/journal.pone.0198086

**Published:** 2018-05-31

**Authors:** Dhandayuthapani Sudha, Srividya Neriyanuri, Ramya Sachidanandam, Srikrupa N. Natarajan, Mamatha Gandra, Arokiasamy Tharigopala, Muthukumaran Sivashanmugam, Mohammed Alameen, Umashankar Vetrivel, Lingam Gopal, Vikas Khetan, Rajiv Raman, Parveen Sen, Subbulakshmi Chidambaram, Jayamuruga Pandian Arunachalam

**Affiliations:** 1 SN ONGC Department of Genetics and Molecular Biology, Vision Research Foundation, Chennai, India; 2 Research scholar, School of Biotechnology, SASTRA University, Thanjavur, India; 3 Elite School of Optometry, Unit of Medical Research Foundation, Chennai, India; 4 Birla Institute of Technology and Science, Pilani, India; 5 Department of Optometry, Medical Research Foundation, Chennai, India; 6 Center for Bioinformatics, Vision Research Foundation, Chennai, India; 7 Department of Vitreo-Retinal Services, Medical Research Foundation, Chennai, India; 8 R.S. Mehta Jain Department of Biochemistry and Cell biology, Vision Research Foundation, Chennai, India; Odense University Hospital, DENMARK

## Abstract

X-linked retinoschisis (XLRS) is a retinal degenerative disorder caused by mutations in *RS1* gene leading to splitting of retinal layers (schisis) which impairs visual signal processing. Retinoschisin (RS1) is an adhesive protein which is secreted predominantly by the photoreceptors and bipolar cells as a double-octameric complex. In general, XLRS patients show wide clinical heterogeneity, presenting practical challenges in disease management. Though researchers have attempted various approaches to offer an explanation for clinical heterogeneity, the molecular basis has not been understood yet. Therefore, this study aims at establishing a link between the phenotype and genotype based on the molecular mechanism exerted by the mutations. Twenty seven XLRS patients were enrolled, of which seven harboured novel mutations. The mutant constructs were genetically engineered and their secretion profiles were studied by *in vitro* cell culture experiments. Based on the secretory profile, the patients were categorized as either secreted or non-secreted group. Various clinical parameters such as visual acuity, location of schisis, foveal thickness and ERG parameters were compared between the two groups and control. Although the two groups showed severe disease phenotype in comparison with control, there was no significant difference between the two XLRS groups. However, the secreted group exhibited relatively severe disease indications. On the other hand molecular analysis suggests that most of the *RS1* mutations result in intracellular retention of retinoschisin. Hence, clinical parameters of patients with non-secreted profile were analyzed which in turn revealed wide variability even within the group. Altogether, our results indicate that disease severity is not merely dependent on secretory profile of the mutations. Thus, we hypothesize that intricate molecular detail such as the precise localization of mutant protein in the cell as well as its ability to assemble into a functionally active oligomer might largely influence disease severity among XLRS patients.

## Introduction

X-linked retinoschisis (XLRS) (OMIM 312700) is a retinal dystrophy, characterized by spoke wheel pattern in the retina, schisis (splitting) within the retinal layers and reduced b-wave amplitude on electroretinogram (ERG) resulting in visual deterioration in affected males [1]. The estimated worldwide prevalence of XLRS ranges from 1:5000 to 1:25000 males [2].

*RS1* (Retinoschisin 1) (OMIM 300839) is the gene implicated in the disorder, which contains six exons spanning a region of 32.4 Kb in the X chromosome [3]. It encodes a 24 KDa protein known as retinoschisin (RS1), which is assembled as a double octamer ring and secreted predominantly by the photoreceptors and bipolar cells of the retina as well as by the pineal gland [4,5]. RS1 plays a major role in cell–cell interactions and cell adhesion, thus preserving the structural and functional integrity of the retina. Retinoschisin is organized into three distinct domains—a signal sequence, RS1 domain and a discoidin domain. The signal peptide guides the translocation of nascent RS1 into the endoplasmic reticulum, the function of the RS1 domain is yet to be explored and recent studies have shown that it is not required for the oligomerization of the protein, while, the discoidin domain contributes to the adhesive function of RS1 [5–7].

XLRS patients show broad mutation spectrum across the entire *RS1* gene, including missense, deletions, duplications, frameshifts and splice site mutations [2]. In general, XLRS patients show diverse phenotype and disease severity which remains unexplained till date [8,9]. Previous reports on phenotype-genotype correlation in XLRS focussed the clinical characteristics and mutation type [10–14]. However, various types of mutations resulted in a similar phenotype showing no significant correlation. Pioneer studies on the molecular mechanism causing XLRS identified that irrespective of the mutation type, most *RS1* mutations affected the secretion of retinoschisin. Therefore, Wang and group speculated that the secretory pattern of each RS1 mutant might determine the severity of the disorder rather than the mutation type [15].

As far as cure for Retinoschisis is concerned, no treatment is available till now to completely arrest the natural progression of schisis formation in the retina. One of the primary reasons behind the lacunae in efficacious disease management is the incomplete knowledge on pathobiology as well as phenotype heterogeneity in XLRS. Hence the molecular mechanism resulting in variable disease severity needs to be understood in order to design effective drugs, combating the symptoms. So, this study aims at investigating the variable disease severity in XLRS patients (Indian cohort) by correlating the secretory pattern of various *RS1* mutations with clinical parameters such as age of the patient, visual acuity, fundus findings, optical coherence tomography and electroretinography. Our findings demonstrated that the secreted group patients exhibited relatively severe disease indications; however the disease severity was not largely dependent on the secretory profile of the mutations.

## Materials and methods

### Clinical examination and sample collection

Twenty seven male patients (related as well as unrelated) clinically diagnosed with retinoschisis were recruited for the study. Written informed consents were obtained from the patient or family members. The entire study protocol was approved by the Vision Research Foundation review board, ethics committee (Ref no. 202-2009-P) and adhered to the tenets of declaration of Helsinki. All the patients underwent standard ophthalmic evaluations which included detailed history, objective refraction, visual acuity and fundus examination using indirect ophthalmoscopy. Color fundus photographs (FF450IR, Carl Zeiss Meditec AG, Jena, Germany), Cirrus high definition optical coherence tomography (OCT) (Carl-Zeiss Meditec AG, Jena, Germany) images using 5-line raster scan (4096 A-scans) protocol and full field electroretinogram (ERG) recordings using Ganzfeld stimulator were documented in only some of the patients due to practical difficulties. ERG was performed as per the International Society for Clinical Electrophysiology of Vision guidelines [16]. Burian-allen contact lens electrodes were used to record the Dark-adapted 0.01 ERG, Dark-adapted 3.0 ERG, Dark-adapted 3.0 oscillatory potentials, Light-adapted 3.0 ERG and Light-adapted 3.0 flicker measurements. Data from both the eyes were considered for the study. Pedigrees were documented and peripheral blood samples were collected from the patient and family members. DNA collected from 50 healthy individuals of south Indian ethnicity was used for the study. The controls were recruited at the vitreoretinal outpatient clinic, Sankara Nethralaya, Chennai, India as a part of another study by adhering to the tenets of Helsinki declaration [17]. All controls were males with a mean age of 50 years. In brief, the individuals were subjected to a thorough eye examination which included subjective refraction, best-corrected visual acuity, slit-lamp examination and fundus evaluation by binocular indirect ophthalmoscopy and stereoscopic evaluation. A detailed family history of medical conditions was collected and individuals with either a past or present history of any retinal disease or abnormalities were excluded.

### Gene sequencing

Genomic DNA was extracted from the peripheral blood of patients using Nucleospin kit (Macherey-Nagel, Duren, Germany) according to the manufacturer’s instruction. Primer sequences for all the six exons of the *RS1* gene and its Polymerase Chain Reaction (PCR) cycling conditions were obtained from the literature [18]. The amplified products were enzymatically purified by treating with *E*. *coli* Exonuclease I and Fast Alkaline phosphatase (Thermo Scientific, Vilnius, Lithuania). The PCR products were bidirectionally sequenced using cycle sequencing kit (Big Dye Terminator v3.0 Ready, Applied Biosystems, Foster City, CA, USA) and then analyzed by ABI PRISM 3100 *Avant* genetic analyzer (Applied Biosystems Inc.). The output was compared with *RS1* reference sequence from the Ensembl database (ENSG00000102104).

### Bioinformatics analysis

Using SIFT and PolyPhen-2 tools, the phenotypic variation contributed by the missense mutations were assessed. For structural analyses, only the discoidin domain of monomeric RS1 was considered for computing the changes, as the RS1 domain is unique and does not share significant sequence homology with the available protein database. The structure of the wild type retinoschisin discoidin domain was modelled using human coagulation factor V (PDBID: 1CZS) as template, as identified by fold recognition using PHYRE server, which falls in line with similar documented study [19]. RS1 discoidin domain sequence was aligned to the template and the corresponding homology model domain was generated using prime module of Schrodinger suite (Schrodinger, LLC, New York, NY, 2016). During the modelling process, the structure was loop refined, geometry optimized and the disulphide bonds were also fixed using Prime. Similarly, the structures of the RS1 mutants were also generated using Prime with modelled RS1 wild type structure as template.

The wild type and RS1 mutant modelled structures were subjected to Molecular dynamics (MD) simulation studies to infer the structural stability. All the predicted structures were found be highly plausible in terms of proper stereochemistry as validated by Ramachandran plot (with no residues in disallowed region). The MD simulation studies of wild type and seven RS1 mutants were performed using Desmond software with all the parameter set based on a similar study reported earlier [14]. The Root Mean Square Deviation (RMSD) for the protein backbone and Root Mean Square Fluctuation (RMSF) of the residues were plotted to analyse the convergence of the structure to equilibrium. The optimal and lowest potential energy structures resulting at the end of the simulation was chosen for further studies. The simulation trajectories were used to assess the protein stability changes.

Further, the changes in hydrophobic surface volume of the wild type and mutants were mapped using inbuilt option in Schrödinger maestro. And, the volume of hydrophobic and hydrophilic surface area (in Angstroms unit) was quantified and tabulated. These calculations predicted the deregulated hydrophobic surface in the mutant structures. Finally, the wild type and mutant structures were analysed for the disulfide bond formation using PDBsum server towards inferring the structural compactness [20].

In a recent study, Cryo-electron microscopic structure of RS1 (PDB ID 3JD6) was resolved (available at RCSB database) and the functionally active wild type RS1 was reported to attain a paired double octameric structure [5]. Hence, besides the monomeric form, we attempted modeling of the multimeric structure of wild type and RS1 mutants. The insertion mutations could not be modelled as they exhibited multiple steric hindrances that were not refined even through iterative minimization processes. Accordingly, only point mutations were modelled using pymol and refined with Molecular Operating Environment (MOE) energy minimization module [21,22]. Further, the hydrophilic and hydrophobic surface areas of the multimeric complex were quantified using Schrodinger maestro, as discussed in the case of monomers.

### Genetic engineering of the mutant constructs

pCMV-Tag4 vector (Stratagene, Santa Clara, CA, USA) cloned with wild type *RS1* cDNA (gift from Dr. Camasamudram Vijayasarathy, NEI, USA) was used as template to create mutant constructs harboring the novel mutations by genetic engineering. *In vitro* mutagenesis was performed to incorporate the missense mutations at the specific site using Quickchange Site-Directed mutagenesis kit (Stratagene, La Jolla, CA, USA). Large duplication, frameshift and nonsense mutations were created by homologous recombination based Gibson assembly Master mix (New England Biolabs Inc., Ipswich, MA, USA) as per manufacturer’s protocol. The respective patient’s genomic DNA was used as template to generate the insert with the desired mutation (frameshift and nonsense mutations) and then cloned with the linearized plasmid DNA as illustrated in S1 Fig. The primer sequences used to introduce the mutations by site directed mutagenesis and Gibson assembly are provided in S1 Table. The clones were then verified by direct sequencing as described earlier.

### Cell culture and western blotting

COS7 cells (obtained from The European Collection of Authenticated Cell Cultures through Sigma-Aldrich, product number 87021302-1VL in 2012) were grown in Dulbecco’s modified Eagle’s medium with 10% fetal bovine serum. A day before transfection, 6X10^5^ cells/well were seeded on to a 6 well plate and serum starved. After 24 hours, the cells were transiently transfected with wild type or the mutant constructs using Fugene HD transfection reagent (Promega, Madison, WI, USA) at a ratio of 2:3, according to the manual’s instruction. Following 4 hours of transfection, the serum starved medium was removed and the cells were supplied with medium containing 10% FBS.

After 72 hours of transfection, the cells and medium fractions were collected separately. Total cell lysate was prepared using radio immunoprecipitation assay (RIPA) buffer (25mM Tris Cl (pH 7.6), 150mM sodium chloride, 1% Triton X, 0.1% Sodium dodecyl sulphate (SDS) and 0.5% Sodium glyoxycholate) and the culture medium was vacuum concentrated. The samples were boiled in laemmli buffer for 10 min at 95°C.

The protein fractions were separated on a 12% SDS- polyacrylamide gel electrophoresis (SDS-PAGE) and the proteins were transferred on to a nitrocellulose membrane and incubated with rabbit monoclonal anti-FLAG antibody (Cell signaling technologies, Danvers, MA, USA, product number #14793) at a dilution of 1:500. After washing with phosphate buffered saline tween-20, the membrane was incubated with the horse radish peroxidase (HRP)-conjugated mouse anti-rabbit secondary antibody (Santa Cruz Biotechnology, Dallas, Texas, USA product number sc-2357-CM) at a dilution of 1: 2000 and then washed again. The samples in the blot were assessed for protein of interest by adding chemiluminescence substrate over the blot and images were captured using chemidoc imaging systems (Bioscreen Instruments, India and Bio-Rad, California, USA).

### Immunocytochemistry

COS7 cells were grown on a coverslip and transfected with the wild type or mutant constructs. After 72 hrs of incubation, cells were fixed with 4% paraformaldehyde for 10 min, permeabilized using 0.2% Tween 20 and blocked for 1 hour with 2.5% bovine serum albumin in phosphate buffered saline (PBS). The cells were then incubated with anti-FLAG primary antibody (1:100) for overnight at 4°C, and then with Alexa fluor 488 labeled goat antirabbit IgG secondary antibody (Thermo Fischer Scientific, Carlsbad, CA, USA) at a dilution of 1:250 for 1 hour at room temperature. Following incubation with primary and secondary antibodies, the cells were washed thrice in PBS containing 0.1% Tween 20. After immunostaining, the cover slips were mounted on slides using Vectashield antifade mounting media with DAPI (Vector Laboratories Inc., Burlingame, CA, USA) and visualized under a fluorescence microscope (Carl-Zeiss Meditec AG, Jena, Germany).

### Statistical analysis

Statistical analysis was performed using Statistical Package for the Social Sciences (SPSS) 17 software. Normality tests showed that the data was not normally distributed (Shapiro wilk, p<0.05). Hence non-parametric One-way ANOVA (Kruskal-Wallis H test) was used to compare ERG parameters between control and two XLRS groups (non-secreted and secreted). Mann-Whitney U test was used for independent comparison between the non-secreted and secreted groups. A p-value cut-off of <0.02 was set as statistical significance, on applying Bonferroni correction.

## Results

### Clinical observation

All the 27 patients presented with clinical features of retinoschisis, however 1 patient (F19/P22) had additional systemic abnormalities such as developmental delay, sensorineural hearing loss and hypotonia [23]. The median value (Inter quartile range (IQR)—25^th^ percentile, 75^th^ percentile) of the age of the patients at the time of presentation was 10 (6, 24) years and the age of onset of symptoms was within their first decade. The patients presented with a moderate visual impairment of median value 0.6 logMAR (range, 0.47, 1.15) and exhibited a hyperopic refractive error of median value +1.75 Diopters (0.50, +4.5). On examining the anterior segment, 4 eyes were found to have cataract, 1 eye was pseudophakic and 1 other eye was aphakic.

**Fig 1:**
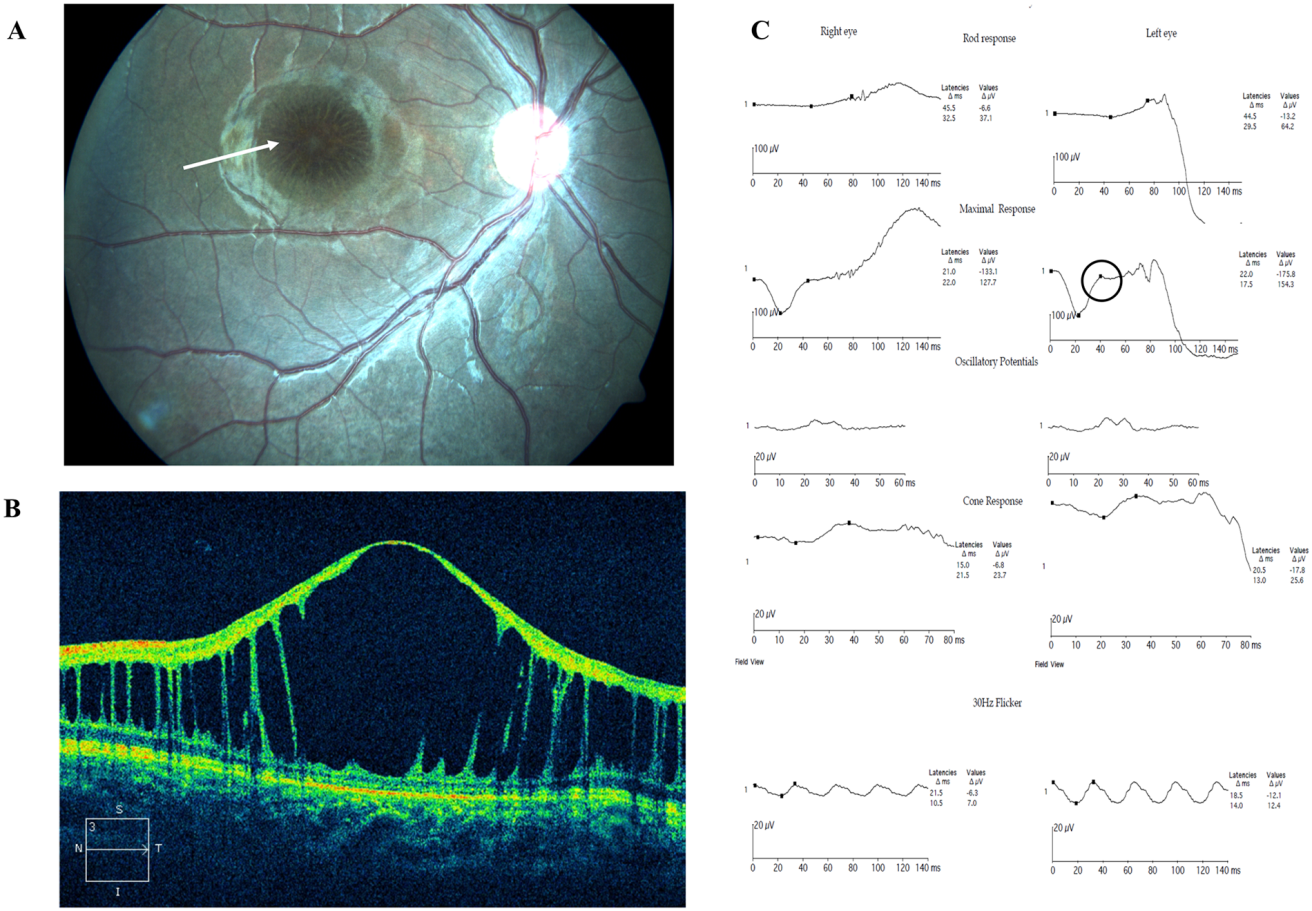
Representative clinical images and data of XLRS patients. (A) Fundus exhibiting spoke wheel pattern like schisis at the macula (indicated by arrow). (B) Optical coherence tomography showing splitting of the inner retinal layers. (C) Electroretinogram showing reduced waveforms of rod and cone responses, a negative b-wave pattern noted on standard combined response (circled).

Based on the location of schisis, the phenotype was classified as either foveal schisis (15 eyes) or foveal + peripheral schisis (39 eyes) wherein 9 eyes had retinal detachment. Tapetal-like metallic reflex of the fundus was observed in 8 eyes and vitreous veils in 5 eyes (Table 1). ERG recordings showed that eyes with foveal + peripheral schisis showed significantly reduced amplitudes when compared to eyes with foveal schisis alone on all scotopic, photopic, standard combined and flicker measurements. OCT analysis showed schisis involving various layers of the retina with elevated foveal contour in 56% of the affected eyes. Detailed phenotype descriptions of the patients have already been reported as a separate study [24]. Representative fundus picture, OCT image and ERG readings of XLRS patients are shown in Fig 1. Analysis of the family history showed that most retinoschisis pedigrees showed a typical X-linked recessive pattern of inheritance, while few were sporadic cases (S2 Fig).

**Table 1:** List of XLRS patients and their clinical characteristics.

FAMILY/ PATIENT	AGE (YR) SEX	SCHISIS OD/OS	ERG OD/OS	OCT	OTHER FINDINGS
F1/P1	4/M	FS+PS/ FS+PS(RD)	EN/EN	S	Cataract
F2/P2	6/M	FS/ FS	Reduced/ Reduced	NT	Vitiligo, Tapetal reflex
F3/P3	9/M	FS/ FS	Reduced/ Reduced	B	Tapetal reflex
F3/P4	7/M	FS/ FS	EN/ Reduced	B	Tapetal reflex
F4/P5	9/M	FS+PS/ FS+PS(RD)	NT/NT	NT	Vitreous veils, cataract
F5/P6	21/M	FS/ FS	Reduced/ Reduced	B	-
F6/P7	40/M	FS+PS/ FS+PS(RD)	EN/EN	B	Epilepsy
F7/P8	25/M	FS+PS/ FS+PS(RD)	EN/NR	S	Cataract
F8/P9	45/M	FS+PS/ FS+PS	Reduced/ Reduced	B	Cataract
F8/P10	41/M	FS+PS/ FS+PS	NT/NT	B	-
F9/P11	14/M	FS+PS(RD)/ FS+PS(RD)	NT/NT	NT	Vitreous veils
F10/P12	9/M	FS+PS/ FS+PS	NT/NT	S	-
F11/P13	7/M	FS+PS/ FS+PS	Reduced/ Reduced	B	Tapetal reflex
F12/P14	27/M	FS+PS/ FS+PS	Reduced/ Reduced	B	-
F12/P15	25/M	FS+PS/ FS+PS	Reduced/ Reduced	B	-
F13/P16	11/M	FS/FS	Reduced/ Reduced	B	-
F14/P17	12/M	FS+PS/ FS+PS(RD)	EN/EN	B	-
F15/P18	19/M	FS+PS/ FS+PS(RD)	NT/NT	S	Vitreous veils, aphakia
F16/P19	26/M	FS+PS/ FS+PS	Reduced/ Reduced	B	Glaucoma
F17/P20	8/M	FS+PS/ FS+PS	EN/EN	S	-
F18/P21	12/M	FS/ FS+PS	Reduced/ Reduced	NT	-
F19/P22	2/M	FS+PS/ FS+PS(RD)	EN/EN	S	Delayed milestone, hearing loss
F20/P23	11/M	FS+PS/ FS+PS	EN/EN	NT	-
F20/P24	39/M	FS+PS/FS+PS	NT/NT	NT	Pseudophakia
F21/P25	20/M	FS+PS/FS	NT/NT	S	-
F22/P26	37/M	FS/FS	Reduced/ Reduced	NT	-
F23/P27	18/M	FS+PS/FS	EN/EN	S	-

YR, years; OD, right eye; OS, left eye; M, male; RD, retinal detachment; FS, foveal schisis; PS, peripheral schisis; R, reduced b-wave; EN, electronegative; NR, non recordable; NT, not tested; B, both eyes; S, single eye.

### *RS1* gene screening

On genotyping the probands, 24 patients were found to have hemizygous mutations in the *RS1* gene, while 3 patients were negative for *RS1* mutation (Table 2). The mutation spectra included missense, duplication, frameshift, nonsense and splice site mutations with missense being the most prevalent type (~58%). Considering only the unrelated patients, 85% (17 patients) had mutations lying in the discoidin domain, whereas few had mutations lying in the leader sequence (2 patients) and in the C-terminal domain (1 patient) of the RS1 protein (S3 Fig).

**Table 2:** *RS1* mutations identified in the study and their secretion profiles.

FAMILY/ PATIENT	MUTATION	AMINO ACID CHANGE	SECRETION	SIFT/PolyPhen-2 ANALYSIS
F1/P1	c.376G>C^†^	D126H	No secretion	Damaging/Probably damaging
F2/P2	c.583_585dupATC^†^	I195dup	No secretion	Cannot be analyzed
F3/P3	c.387_434dup48^†^	Q129_I144dup	No secretion	Cannot be analyzed
F3/P4	c.387_434dup48^†^	Q129_I144dup	No secretion	Cannot be analyzed
F4/P5	c.579delC^†^	I194Sfs*43	No secretion	Cannot be analyzed
F5/P6	c.374T>G^†^	I125R	No secretion	Damaging/Probably damaging
F6/P7	c.590G>A	R197H	No secretion	Damaging/Probably damaging
F7/P8	c.286T>C^#^	W96R	No secretion	Damaging/Probably damaging
F8/P9	c.421C>T^#^	R141C	No secretion	Damaging/Probably damaging
F8/P10	c.421C>T^#^	R141C	No secretion	Damaging/Probably damaging
F9/P11	c.663dupC^†^	K222Qfs*42	Very mild secretion	Cannot be analyzed
F10/P12	C.544C>T^#^	R182C	Very mild secretion	Damaging/Probably damaging
F11/P13	c.422G>A^#^	R141H	Secreted	Tolerated/Probably damaging
F12/P14	c.422G>A^#^	R141H	Secreted	Tolerated/Probably damaging
F12/P15	c.422G>A^#^	R141H	Secreted	Tolerated/Probably damaging
F13/P16	c.214G>A^#^	E72K	Secreted	Tolerated/Probably damaging
F14/P17	c.214G>A^#^	E72K	Secreted	Tolerated/Probably damaging
F15/P18	c.214G>A^#^	E72K	Secreted	Tolerated/Probably damaging
F16/P19	c.421C>A^#^	R141S	Secreted	Tolerated/Possibly damaging
F17/P20	c.349C>T^†^	Q117*	Null expression	Cannot be analyzed
F18/P21	c.267T>A^#^	Y89*	Null expression	Cannot be analyzed
F19/P22	c.78+1G>T	p.?	No data	Cannot be analyzed
F20/P23	c.78+1G>T	p.?	No data	Cannot be analyzed
F20/P24	c.78+1G>T	p.?	No data	Cannot be analyzed
F21/P25	No mutation	-	-	-
F22/P26	No mutation	-	-	-
F23/P27	No mutation	-	-	-

^†^ Novel mutations identified in the study;

^#^ Data obtained from literature [19,25,26])

Totally, 7 novel *RS1* mutations were identified in the study and control screening was performed in 50 samples. Co-segregation analysis was carried out in families where the parent’s or siblings’ samples were available. All the validated novel mutations were submitted to dbSNP, NCBI database of Genetic Variation (http://www.ncbi.nlm.nih.gov/SNP/snp_viewBatch.cgi?sbid=1056549) and Leiden Open Variation Database (https://grenada.lumc.nl/LSDB_list/lsdbs/RS1).

### *In silico* analysis

The damaging effect of all missense mutations were assessed using bioinformatics tools such as SIFT and PolyPhen-2 (Table 2). Further, to examine the changes caused by mutations at the structural level, *in silico* approaches such as homology modelling and molecular dynamic simulations were employed. Only 4 of the novel mutations (D126H, I195dup, Q129-144dup and I125R) were studies by *in silico* analysis as the sequences of nonsense and frameshift mutants could not be matched against any ideal template structure. The other reported mutations were not considered for the analysis as they have already been studied [14,19]; however, 3 reported mutations (W96R, E72K and R197H) were used as reference.

**Fig 2:**
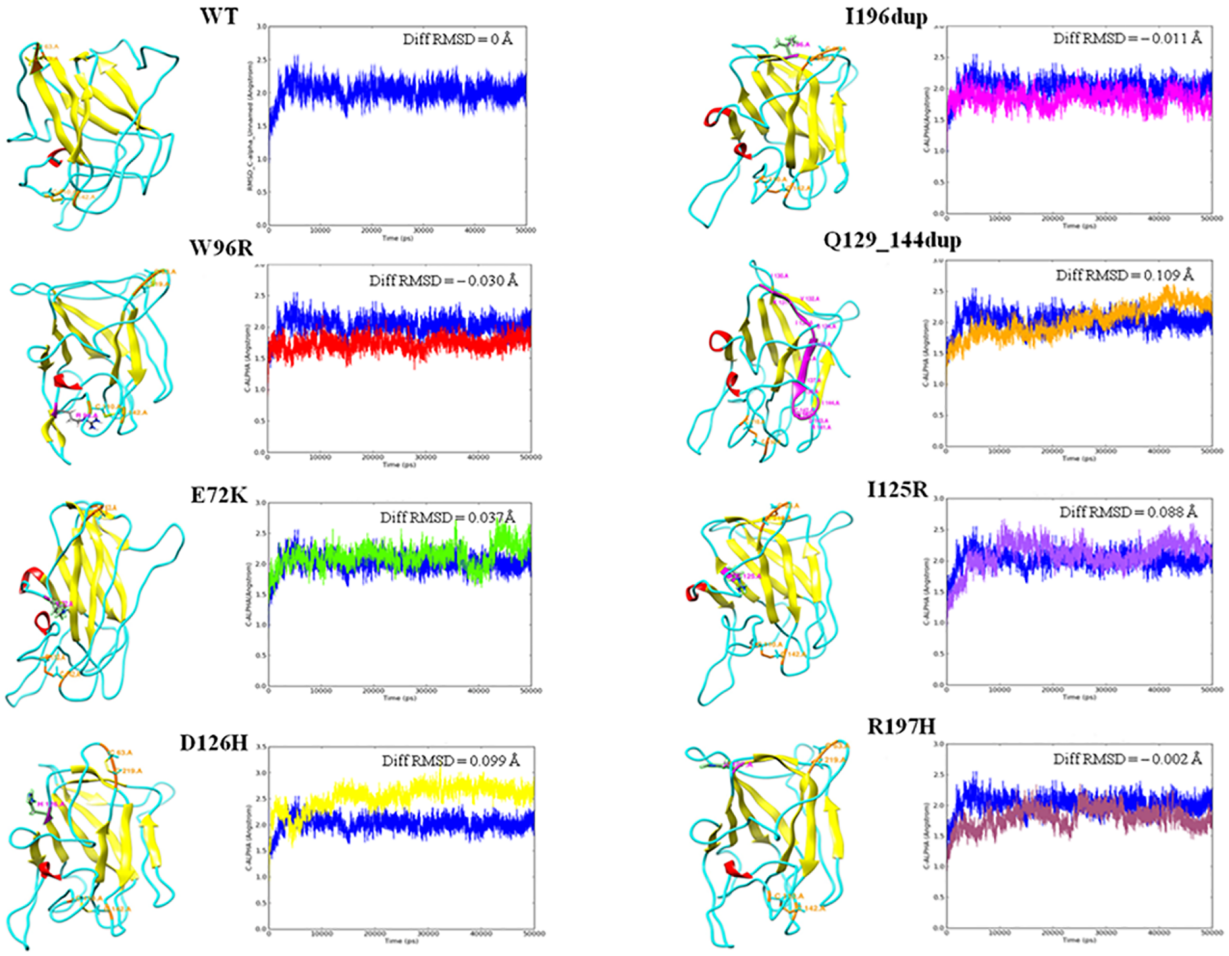
Tertiary structure and RMSD graph of WT and RS1 mutants inferring differences in backbone stability. In the tertiary structure, helices are shown in red, sheets in yellow, loops in cyan, mutation spots in magenta with the residue label and disulphide bonds in orange colour. Diff RMSD value (Å) specified in each graph represents the average difference in the RMSD value (Å) of every mutant in comparison to the wild type.

The core structure of the RS1 discoidin domain provided a base for the 3D mapping of the identified pathological mutations spread across the discoidin domain. The protein backbone RMSD trajectories of the modeled proteins during the MD simulation are shown in Fig 2. The wild type protein was found to be stabilized at an RMSD value of around 0.145 Å. Higher deviations in mutants E72K (0.182 Å), D126H (0.244 Å), Q129_144dup (0.254 Å) and I125R (0.233 Å) infer gain of backbone flexibility and loss of stability in comparison to the WT. The mutants with comparably lower deviations namely, W96R (0.115 Å), I195dup (0.134 Å) and R197H (0.143 Å) infer gain of backbone rigidity. These perturbations in backbone RMSD of mutants can be directly attributed to the effect of mutations in modulating the structure-function relationships. Moreover, high residue fluctuations were also observed in the RMSF plots of mutants reinforcing the structural changes (data not shown).

Secondary structure changes during the MD simulation were also predicted to infer the impact of mutations at the fold level (Table 3). The results infer that alpha helical structures in all the mutant structures were consistent, except for R197H showing a negligible difference. However, the proportion of beta strand showed a significant decrease in E72K, Q129_144dup and I125R whereas a significant gain in I195dup mutant, in comparison with the WT. Such secondary structure element changes might affect the stabilization of the overall tertiary structure formation and in turn affect the functional aspects of the proteins. The modeled WT RS1 had two intact disulfide bonds while Q129_144dup resulted in loss of one disulfide bond. On the other hand, all other mutants had an increase in the intra disulfide bridge distance which might confer changes at functional level.

**Table 3:** Structural effects of various monomeric *RS1* mutations as inferred by *in silico* analysis.

RS1	% OF HELIX DURING MDS	% OF STRAND DURING MDS	HYDROPHILIC AREA (Å)	HYDROPHOBIC AREA (Å)	NUMBER OF DISULFIDE BONDS
WT	0.00	35.95	7163.845	707.130	2 (Cys63-Cys219, Cys 110-Cys142)
W96R	0.00	35.89	7594.86	517.153	2 (Cys63-Cys219, Cys 110-Cys142)
E72K	0.00	34.24	7599.401	479.494	2 (Cys63-Cys219, Cys 110-Cys142)
D126H	0.00	35.88	8392.535	600.462	2 (Cys63-Cys219, Cys 110-Cys142)
I196dup	0.00	36.68	7918.714	549.501	2 (Cys63-Cys220, Cys 110-Cys142)
Q129_144dup	0.00	30.61	8599.731	648.115	1 (Cys110-Cys158)
I125R	0.00	34.10	8134.669	548.585	2 (Cys63-Cys219, Cys 110-Cys142)
R197H	0.01	35.69	7640.360	471.839	2 (Cys63-Cys219, Cys 110-Cys142)

MDS, molecular dynamic simulation.

Further, the overall hydrophobic and hydrophilic surface area for each protein was calculated and tabulated. When the hydrophobicity of a mutant is altered in comparison to WT, it could have a direct impact on the functionality of the protein. Based on these calculations, a reduction in hydrophobic surface value and an increase in hydrophilic surface value were observed in all the mutants in comparison to WT (Table 3). The *in silico* analyses were interpreted only with respect to the monomeric form of retinoschisin.

**Fig 3:**
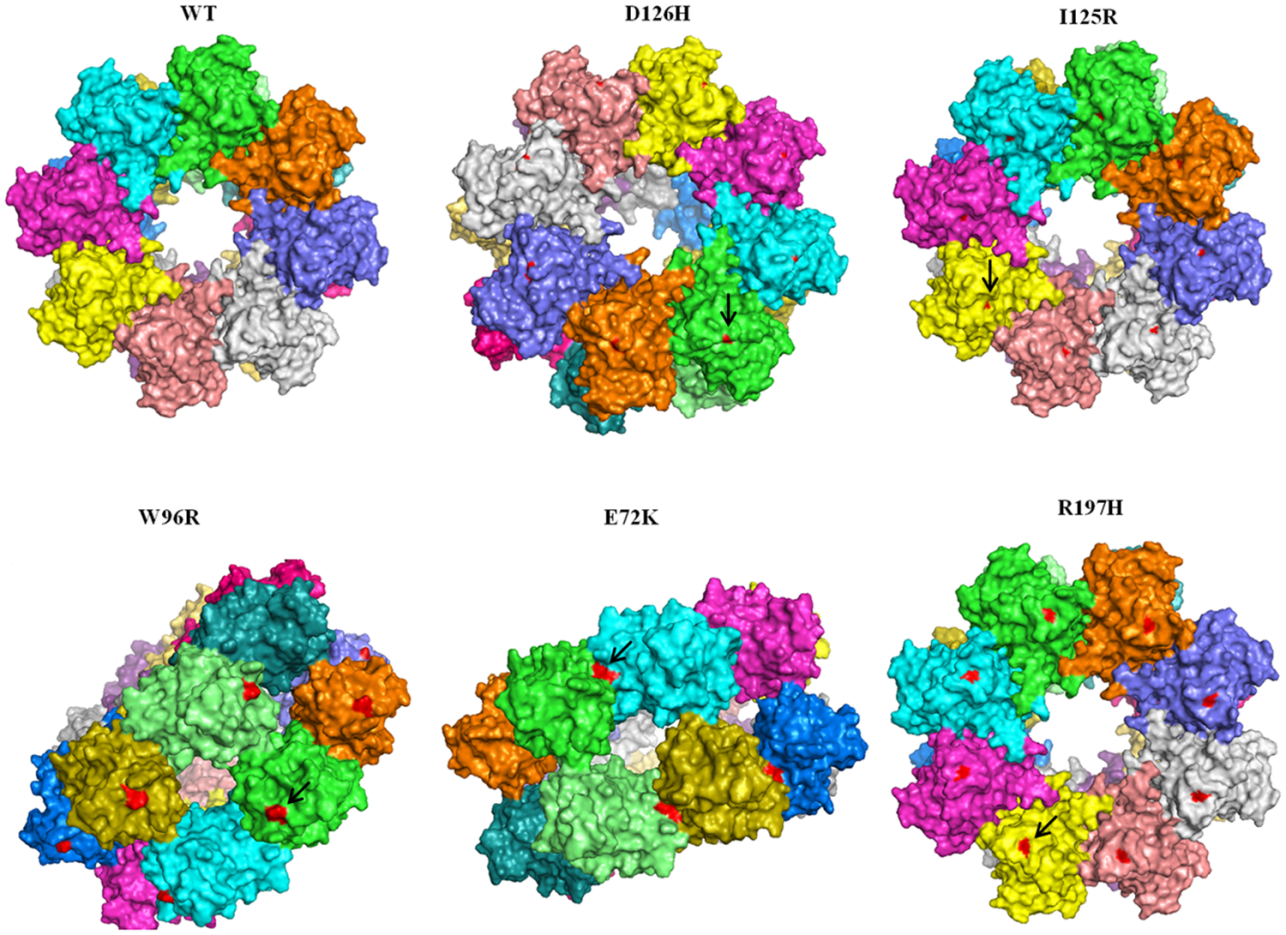
Double octameric structure of wild type retinoschisin and its mutants visualized using pymol. The red coloured regions (represented by an arrow) indicate the mutation points in each mutant structure.

On analyzing the multimeric structures of wild type and mutants (W96R, E72K, R197H, D126H and I125R), all mutation points were found exposed on the surface area of discoidin domain, except E72K, which spanned the interacting interface of the domains as reported by Ramsay and group (Fig 3) [27]. The hydrophobic ratios of multimeric structures were found to be similar and comparable to the monomeric forms. However, the hydrophilic ratios varied, especially in the case of D126H and R197H (Table 4). This variation in the hydrophobic surface area of multimeric forms might have an impact on the structure functional relationship of the mutant proteins in terms of cell adhesion, secretion and other intermolecular interactions.

**Table 4:** Hydrophobic and hydrophilic effects of multimeric *RS1* mutant structures inferred by *in silico* analysis.

RS1	HYDROPHOBIC AREA (Å)	HYDROPHILIC AREA (Å)
WT	16070.820	133919.441
E72K	15600.883	134705.587
W96R	15354.914	136430.290
R197H	15832.470	133087.812
D126H	15445.807	133848.462
I125A	15847.963	134624.914

K222Qfs*42 and I194Sfs*43 are frameshift mutations, extending beyond the termination point of retinoschisin, caused by duplication and deletion of a single nucleotide respectively. Typically, Cys 219 is involved in an intramolecular disulphide bond with Cys63 and a mutation disrupting this bond is known to cause protein misfolding; while, Cys223 is responsible for intermolecular disulphide bond formation with Cys59 and is essential for the oligomerization of WT RS1 [25]. Based on sequence analysis, Cys223 in mutant K222Qfs*42 was found to be shifted to position 224, however, the extended frameshift did not constitute any additional cysteine residues. Whereas, in mutant I194Sfs*43 the frameshift resulted in loss of Cys219 and Cys223, but, introduced 2 new cysteine residues at position 216 and 227. These *in silico* observations indicated that these (novel) *RS1* mutants were most likely pathogenic.

### Analysis of secretion pattern of RS1 mutants

The secretory profile of all the reported RS1 mutants in our sample cohort have already been characterized by various groups worldwide [19,25,26]. Therefore, only the novel mutants and 1 reported mutant (R197H that was not characterized) were taken for the analysis. These include 3 point substitutions (I125R, D126H, R197H), 1 nonsense mutation (Q117*), 2 duplications (I195dup, Q129_144dup) and 2 frameshift mutations (K222Qfs*42, I194Sfs*43). These mutant constructs were genetically engineered and then transfected into COS7 cells. The total cell lysate served as the intracellular fraction, while the culture medium in which the cells were grown served as the secretory fraction.

**Fig 4:**
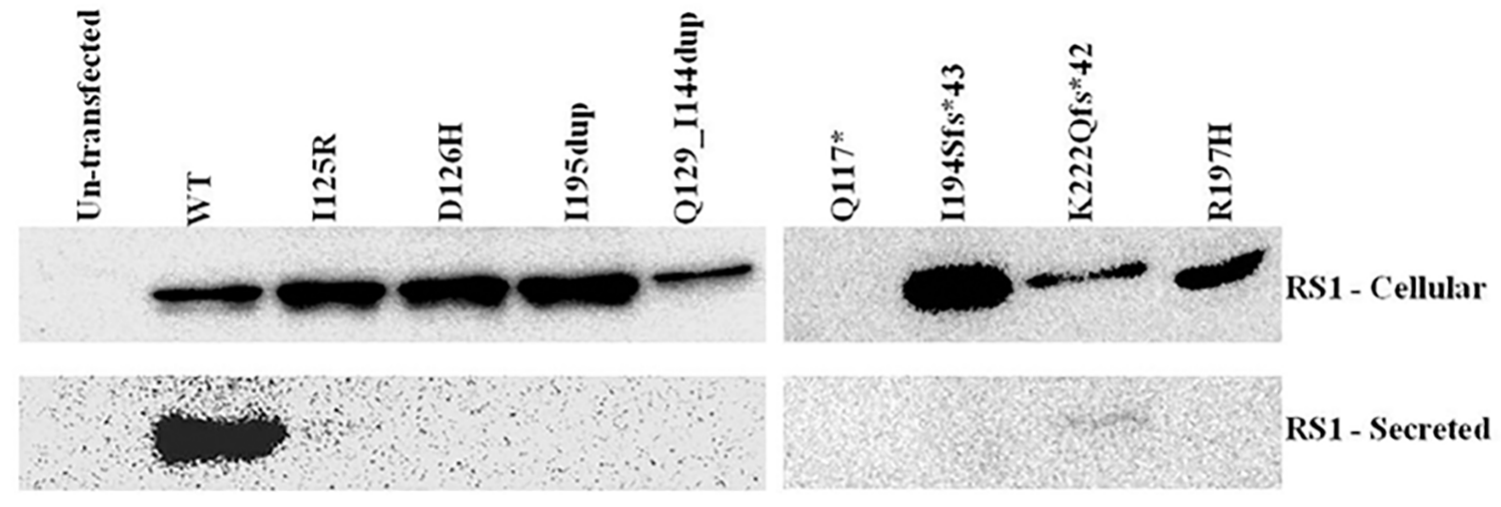
Immunoblot analysis of WT and RS1 mutants in cellular and secreted fractions of COS7 cells. RS1 was detected using anti-FLAG antibody after 72 hours of transfection. Un-transfected lane served as negative control.

Wild type (WT) RS1 protein was detected in the intracellular as well as in the medium fraction, whereas most of the mutants were detected only in the intracellular fraction. Exception to this is K222Qfs*42, which showed a very mild extracellular secretion although its intracellular fraction retained a major portion of the mutant protein. Q117*, being a nonsense mutation exhibited null expression (Fig 4), though the mutant mRNA was detected in transfected cells (data not shown). All the mutations characterized in this study lie in the discoidin domain, while K222Qfs*42 is the only mutation located in the C terminal region of the RS1 protein. These results suggest that, regardless of the mutation type, RS1 mutants predominantly exhibit complete intracellular protein retention, affecting the structural organization of the retinal layers.

The molecular weight of the monomeric form of WT RS1 protein was 24 KDa as observed in denaturing and reducing SDS-PAGE. The two frameshift mutations, K222Qfs*42 and I194Sfs*43 created a new reading frame downstream of the original stop codon, resulting in an aberrant protein of molecular weight 28 KDa and 25 KDa respectively. Mutants I195dup and Q129_144dup are two duplication mutations that did not result in any codon frameshift. I195dup is a small duplication inserting 3 nucleotides, while Q129_144dup results from a large duplication consisting of 48 base pairs and was detected at a molecular weight of 26 KDa, corresponding to the added extra codons. The protein products of all the missense mutants were similar to that of WT RS1. The cDNA sequences of all mutants are provided in S2 Table. Un-transfected condition served as negative control for the experiment. The observations were consistent when the experiments were repeated three times.

**Fig 5:**
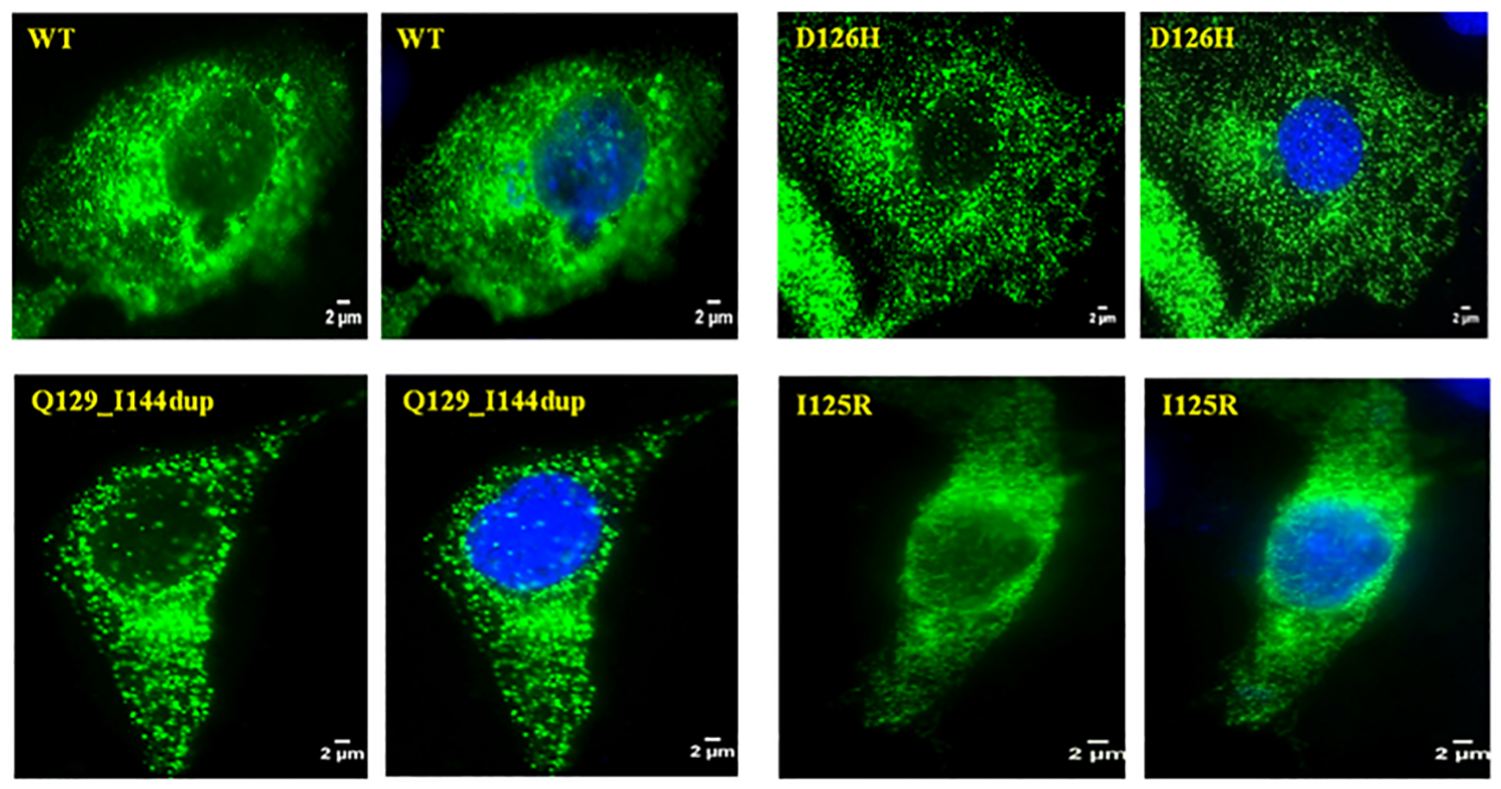
Immunocytochemistry displaying expression and distribution of WT RS1 and various non-secreted RS1 mutants. RS1 immunoreactivity (anti-FLAG antibody) is shown in green while the nucleus is stained blue using DAPI.

As previous studies have shown misfolded mutant proteins to be primarily retained within the ER [15,26] we examined the distribution and accumulation of 3 mutant proteins (D126H, I125R and Q129_144dup) within the cell by immunocytochemistry. On visualization, RS1 immunoreactivity was detected across the entire cytoplasm, almost spreading to the periphery of the cell as seen in Fig 5. None of the 3 tested mutants showed any remarkable accumulation in the ER. Though the mutants were secretion incompetent, there was no significant difference in the pattern of RS1 distribution between the mutants and WT.

### Phenotype-genotype correlation

To perform phenotype-genotype correlation, the patients were categorized into two independent groups based on the secretion profile of mutant RS1 proteins as secreted group (9 patients, 18 eyes) and non-secreted group (10 patients, 20 eyes). Patients who did not show any mutation in the *RS1* gene and those who either harboured a nonsense or splice site mutation were excluded. The secretory profiles of all the reported *RS1* mutations identified in this study were obtained from the literature (Table 2). Phenotype characterization was done based on fundus appearance, OCT images and ERG recordings. All the 19 patients had age of onset of the symptoms within the second decade of their life. The median (IQR, 25^th^ percentile, 75^th^ percentile) value of the age of the patients at the time of presentation was 10.5 years in the non-secreted group and 12 years in the secreted group.

Based on fundus examination, the location of schisis was determined in each group. Twelve (60%) out of twenty eyes in the non-secreted group had schisis involving the foveal as well as peripheral retina, of which four eyes (20%) exhibited retinal detachment; while in the remaining eight eyes (40%) only foveal schisis was noted. Likewise, in the secreted group, sixteen (88%) out of eighteen eyes had foveal and peripheral schisis, of which four eyes (22%) showed retinal detachment; while the other two eyes (11%) had only foveal schisis (Table 5). OCT parameters showed that the secreted group exhibited a relatively decreased foveal thickness (median value) compared to the non-secreted group.

**Table 5:** Comparison of ocular features between non-secreted and secreted XLRS groups.

CLINICAL PARAMETER	NON-SECRETED GROUP (N = 20 eyes)	SECRETED GROUP (N = 18 eyes)
	**Median (IQR)**	**Median (IQR)**
Age	10.5 (6.75, 29)	12 (10, 25.5)
Visual acuity (logMAR)	0.5 (0.47, 1.15)	0.7 (0.6, 1.15)
Foveal thickness (μm)	433 (385, 479)	371.5 (300.5, 486)
	**Number of eyes**	**Number of eyes**
Only Foveal schisis	8	2
Foveal + peripheral schisis	12 (4 eyes had RD)	16 (4 eyes had RD)

IQR, inter Quartile Range (25^th^ percentile, 75^th^ percentile); RD, retinal detachment.

With respect to ERG parameters, only 16 eyes in the non-secreted group and 12 eyes in the secreted group were considered as the ERG readings were either not available or non recordable in the other eyes. Accordingly, the ERG readings from 16 eyes of age matched normal individuals were taken for the analysis. On comparison with control values, the implicit times of all dark-adapted and light-adapted responses were more delayed in secreted group than in the non-secreted group. Although there was a significant reduction in the amplitudes of all dark and light-adapted responses in both the XLRS groups, scotopic b-waves were found to be more reduced in the non-secreted group; whereas in the secreted group, photopic b-wave was more affected. In addition, scotopic and photopic a-waves were noted to be reduced in the secreted group in comparison to the non-secreted group (Table 6). However, irrespective to the control values when compared between secreted and non-secreted group, none of the ERG readings were significantly varying, except for delayed implicit time in dark-adapted 3.0 b-wave noted in the secreted group (p = 0.001, Mann-Whitney U test (data not shown)).

**Table 6:** Comparison of ERG parameters between control samples and XLRS patients showing non-secreted or secreted or protein profile.

ERG PARAMETERS	CONTROL GROUP (N = 16 eyes) Median (IQR)	NON-SECRETED GROUP (N = 16 eyes) Median (IQR)	SECRETED GROUP (N = 12 eyes) Median (IQR)	p VALUE*
**Amplitudes (μV)**				
Dark-adapted 0.01 b-wave	295.1 (269.5, 337.6)	70.4 (16.9, 114.6)	73.9 (40.1, 104.9)	<0.0001
Dark-adapted 3.0 a-wave	208.9 (165.5, 257.5)	170.7 (76.1, 220.4)	139.2 (95.4, 195.7)	0.037
Dark-adapted 3.0 b-wave	483.6 (442.5, 524.7)	168.3 (72.1, 249.4)	170.2 (141.2, 220.7)	<0.0001
Light-adapted 3.0 a-wave	27 (25.1, 31.2)	19.4 (12, 25.3)	17.7 (11.6, 23.7)	0.001
Light-adapted 3.0 b-wave	95.2 (68.4, 135.1)	40.2 (19.8, 57.6)	31.6 (24.2, 45.9)	<0.0001
Light-adapted 30 flicker 3.0 b-wave	60.4 (50.2, 82.0)	25.9 (9.4, 35.0)	18.6 (12.2, 23.5)	<0.0001
Dark-adapted b/a ratio	2.4 (2.0, 2.7)	1.1 (0.9, 1.3)	1.2 (1.0, 1.6)	<0.0001
Light-adapted b/a ratio	3.4 (2.4, 5.1)	2.3 (1.5, 2.7)	2.3 (1.5, 2.9)	0.003
**Implicit times (ms)**				
Dark-adapted 0.01 b-wave	62.8 (59.6, 69.4)	76.5 (71, 80.5)	77.3 (74.4, 82.8)	<0.0001
Dark-adapted 3.0 a-wave	17.5 (16.5, 17.9)	18.5 (17, 20.9)	20.3 (19.5, 21.8)	<0.0001
Dark-adapted 3.0 b-wave	43.3 (42.1, 44.9)	39.3 (35.6, 41.9)	50.3 (46.6, 54)	<0.0001
Light-adapted 3.0 a-wave	16.8 (16.5, 17.5)	18.5 (17.5, 20.0)	19.3 (17, 20)	0.003
Light-adapted 3.0 b-wave	27.5 (27, 28.4)	30 (28.5, 33.9)	31.3 (29.5, 33.5)	<0.0001
Light-adapted 30 flicker 3.0 b-wave	25 (24.5, 25.5)	29.5 (28, 31.9)	29.5 (28.1, 31.6)	<0.0001

μv, microvolts; ms, milliseconds; IQR, inter quartile range (25^th^ percentile, 75^th^ percentile).

*p value = 0.02 (bonferroni correction)

Further, to understand phenotype variability among patients showing same secretion profile, we analyzed the data of 10 patients with non-secreted RS1 profile. It was intriguing to note that there was large phenotype variability in terms of visual acuity, schisis involvement and ERG b/a ratio even among patients with similar age (Table 7). This finding contradicts the hypothesis proposed by Wang et al that disease severity depends on the secretion profile of the mutation [15].

**Table 7:** Clinical data of XLRS patients showing non-secreted RS1 profile.

FAMILY/ PATIENT	EYE	VISUAL ACUITY	REFRACTIVE ERROR	FUNDUS FINDINGS	b/a RATIO—ERG
F1/P1	OD	6/38	5.5	FS+PS	0.82*
	OS	CF at 1 meter	7.5	FS+PS (RD)	0.99
F2/P2	OD	6/24	2.5	FS	1.23
	OS	6/24	1.25	FS	1.28
F3/P3	OD	6/12	2	FS	1.14
	OS	6/12	0.75	FS	1.11
F3/P4	OD	6/18	2.75	FS	0.96
	OS	6/18	2.75	FS	1.09
F4/P5	OD	CF at 3 meters	11.5	FS+PS	Not tested
	OS	Perception of Light		FS+PS (RD)	Not tested
F5/P6	OD	6/18	0.75	FS	1.21
	OS	6/18	0.75	FS	1.3
F6/P7	OD	6/60	8	FS+PS	0.94*
	OS	6/18	8	FS+PS (RD)	0.97*
F7/P8	OD	6/18	5.5	FS+PS	0.75*
	OS	No Perception of light	not measurable	FS+PS (RD)	Non recordable
F8/P9	OD	3/60	-14	FS+PS	1.6
	OS	6/24	-0.75	FS+PS	1.34
F8/P10	OD	6/45	-6	FS+PS	Not tested
	OS	6/18	-1	FS+PS	Not tested

OD, right eye; OS, left eye; CF, counting fingers; Refractive error in diopters, FS, foveal schisis; PS, peripheral schisis; RD, retinal detachment;

*Electronegative wave form.

## Discussion

### Clinical considerations

Fundus examination showed that majority of the affected eyes (~55%) showed schisis in both the foveal as well as peripheral retina and about 1 in 4 patients exhibited retinal detachment. “Most patients had schisis in inner nuclear layer, while few eyes had schisis extending into the inner plexiform layer and inner nuclear layer. Of note, peripheral schisis involved the ganglion cell layer, while foveal schisis involved the inner nuclear and outer retinal layers. Structural alterations like photoreceptor thinning, inner segment-outer segment defects along with atrophy and alterations of retinal pigment epithelium were also observed” [24]. ERG showed reduction in amplitudes of either ‘a’ or ‘b’ wave or both under scotopic or photopic conditions in different eyes. ERG amplitudes were much affected in eyes involving peripheral schisis when compared to eyes with only foveal schisis [24]. The clinical presentations clearly indicate that the disease severity in terms of fundus changes, OCT parameters and ERG readings greatly vary among patients (same ethnicity), iterating phenotype variability.

### Molecular considerations

Based on bioinformatics analyses, *RS1* mutations were found to cause structural perturbations in the protein which were predicted to affect its secretion. A recent report on structural analysis of RS1 has shown wild type RS1 as a paired back-to-back octameric ring, which form the structural basis for its functional role. Further, it is suggested that this molecular model may help in understanding the disruptive effect of many disease-related mutants as it may involve residues that are crucial for assembly of the oligomer [5]. This is evident from our own findings and that of Ramsay and group, where some of the mutants (D126H, I125R, E72K and I195) are shown to be located in the intraoctamer region, which might affect the hexadecamer assembly [27]. However, in the current study, we were not able to assess the multimeric conformation of all the mutants as extensively performed for the monomeric forms which include MD simulations due to the lack of high end computational hardware. But we have performed the basic modelling and hydrophobicity analysis for the multimeric forms with the available resources.

Our molecular characterization studies and those of others have shown most RS1 mutants to be retained within the cell while few mutants showed a mild secretion [15,19,25,26]. As a separate study, schisis fluid accumulated within the intraretinal cavities of XLRS patient 1 and 7 were analyzed by high resolution mass spectrometry. Retinoschisin was not detected in schisis fluid of both the patients suggesting that RS1 was not secreted out by the retinal cells in these patients [28]. This observation correlates with our *in vitro* molecular characterization finding where the mutations harbored by the two patients were shown to be intracellularly retained. Moreover, our bioinformatics analysis deduced an increase in hydrophilic surface area of the novel mutants which might affect its secretory process. This bioinformatics prediction coincides with our *in vitro* findings, as the mutants were not secreted out of the cell. This led to the understanding that lack of RS1 secretion could be the principal pathological mechanism underlying XLRS. Therefore patients with mutations leading to total intracellular RS1 retention would be expected to show a severe disease phenotype. To assess this inference, the clinical parameters of all patients falling under either secreted or non-secreted category were studied in detail.

### Phenotype-genotype relationship

As age might act as a confounding factor in determining the severity of the disorder, we performed Spearman’s correlation analysis to establish the association between age of the patients and the ERG b/a-wave ratio. But, no significant correlation (Spearman’s rho = 0.11, p = 0.6 for the left eyes; Spearman’s rho = 0.31, p = 0.2 for the right eyes) was observed in this cohort. Moreover, we were not able to apply age adjustment while performing statistical analyses due to the small sample size.

All clinical parameters of the two XLRS groups showed remarkable difference in comparison with control values, but, there was no significant difference between the two XLRS groups. However, it is noteworthy that the secreted group exhibited relatively severe disease phenotype with regard to visual acuity (logMAR), ERG (scotopic a-wave and all photopic responses) and OCT parameters (foveal thickness and schisis). Although the full field ERG characteristically showed reduced b-wave amplitude with a normal a-wave, reduction in a-wave has been reported in cases of extensive peripheral schisis or peripheral pigmentary changes [29]. Similarly, in our study, reduced a-wave was observed in several non-secreted as well as secreted group eyes (as majority of the eyes showed schisis involving the peripheral retina), though more commonly observed in secreted group eyes (S4 Fig). Besides, there was wide variability in the phenotype even among patients showing non-secreted RS1 profile. This striking clinical heterogeneity in XLRS causes ambiguity in arriving at a correlation based on the secretion profile of the mutant protein.

Tapetal-like metallic reflex of the fundus is usually associated with Oguchi disease and fundus albipunctatus due to excessive extracellular K^+^ released from the activated neurons [30]. It is rarely seen in retinoschisis and its functional significance is not fully understood [31]. Iannaccone and group hypothesized that the metallic reflex seen in XLRS patients might be due to the intracellular retention of RS1 which was correlated with the phenotype and genotype of a patient harbouring the mutation W112C [32]. However, their speculation contradicts the fact that the metallic reflex is not observed in other XLRS patients carrying the same mutation. In our cohort, the tapetal-like reflex was observed in 4 XLRS patients (F2/P2, F3/P3, F3/P4 and F11/P13). Molecular characterization studies showed 3 of these mutants to be non-secreted while one mutant to be secreted (Table 2). Notably, in our study, this reflex was not noted in other XLRS patients harbouring the same mutation. Thus, it is understood that the tapetal-like reflex in XLRS condition is neither mutation dependent nor due to intracellular RS1 retention, but some unknown mechanism which is yet to be explored. Patient F17/P20 (8 years old) harboring the nonsense mutation, Q117* (c.349C>T) exhibited bullous retinoschisis, small intra cystic hemorrhage and RPE mottling. Molecular characterization of this mutation showed null RS1 expression. Yet, he had an apparently stable fundi and vision in both the eyes during his follow-up evaluation over a period of 7 years; raising the question whether the disease phenotype is fully consistent with a reduction in retinoschisin level. This observation is similar to the report from another XLRS family with 6 affected males (showing RS1 null expression), wherein the young boys had less severe phenotype than the affected older members; indicating that the complete absence of retinoschisin might be substituted by other putative cell adhesion proteins, altering protein-protein interactions during the early stages of the disease [33]. Proteins such as Na^+^ /K^+^ ATPase, SARM1, alpha B crystalline and L-type voltage-gated calcium channel are known to interact with RS1 [7,34,35], however, the structural basis of these interactions and their effect on the disease state has not been established yet. Further, a recent report has shown the existence of a genetic modifier (Tyrosinase) that play an important role in determining the severity of schisis in a retinoschisis mouse model [36]. But, its role and effect in humans have not been studied so far. Therefore, it is also postulated that the presence of variations in RS1 interacting molecules or genetic modifiers of RS1 may potentially modulate disease severity in the respective patient.

### Hypothesis

Based on the study sample comprising patients of Indian ethnicity, it was found that the disease severity in XLRS subjects varied greatly even among those showing the same RS1 secretion profile. This observation adds new information to the earlier report by Wang and group proposing that the disease severity is dependent on the secretory profile of the mutant [15].

The secretory profile of most of the RS1 mutants were secretion incompetent which raised a question on phenotype heterogeneity demonstrated by the non-secreted mutants. Therefore, we attempted co-localization studies to determine the subcellular localization of one of the non-secreted mutants (Q129_144dup). We used N-type calcium channel protein, golgin coiled-coil protein and protein disulfide-isomerase as markers to indicate co-localization at plasma membrane, golgi apparatus and endoplasmic reticulum, respectively. Preliminary data showed overlapping signals of mutant RS1 with N-type calcium channel protein, suggesting plasma membrane localization of the mutant (S5 Fig). It is interesting to compare our results with the findings of Wang and group where they have shown two of the non-secreted mutants (L12H, R102W) to be retained within Endoplasmic reticulum and Golgi apparatus, failing to be transported to the plasma membrane of the cell [15]. However, it is necessary to substantiate our finding by examining the other non-secreted mutants using high resolution microscopy. Structural analysis by *in silico* tools revealed a considerable difference in the hydrophobic and hydrophilic surface area of the non-secreted mutants (Tables 3 and 4). Each mutant was found to display various structural alterations though they exhibited the same secretion profile.

**Fig 6:**
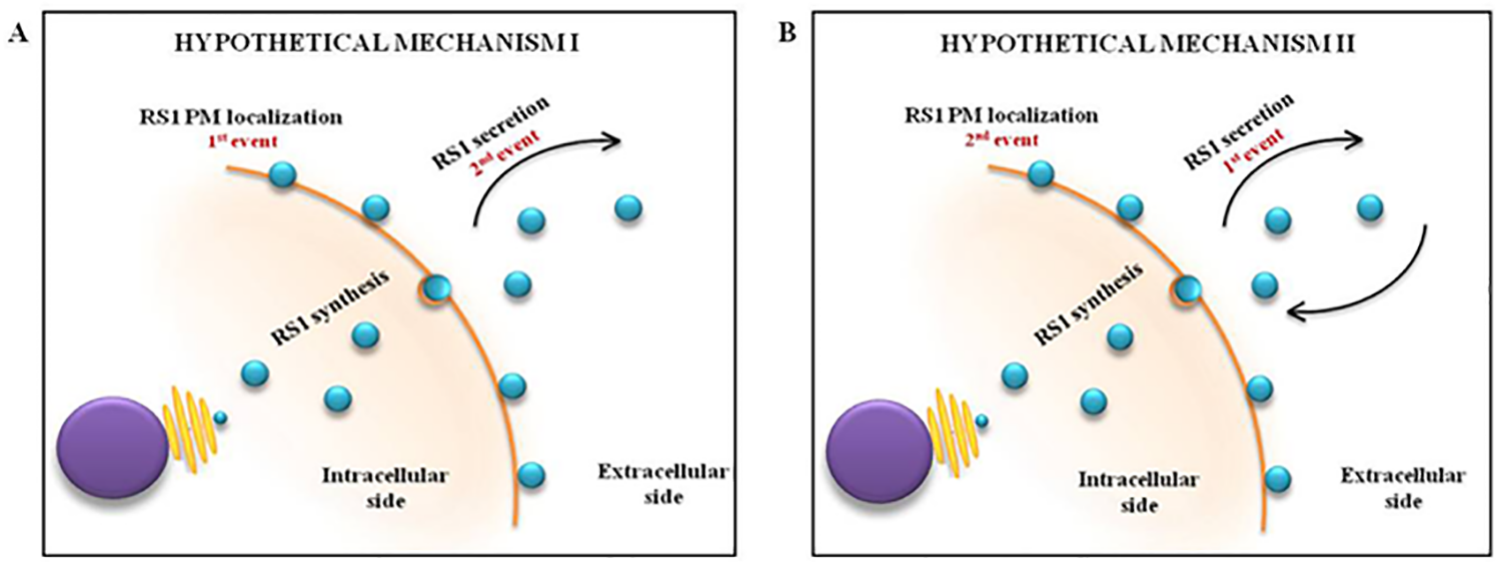
Picture illustrating the hypothetical secretory phenomenon of RS1. (A) Hypothetical mechanism I showing plasma membrane localization of RS1 as the primary event, followed by its secretion into the extracellular side. (B) Hypothetical mechanism II showing extracellular secretion of RS1 as the primary event, followed by its localization to the plasma membrane.

The secretory phenomenon of wild type retinoschisin is still enigmatic. Though RS1 is known to be a soluble secretory protein having affinity to binding to the peripheral membrane of cell [37], only little is known about the sequence of events at play. That is, whether RS1 initially localizes to the peripheral side of plasma membrane followed by its secretion into the extracellular matrix upon receiving cell signals or vice versa, is not yet known (Fig 6). Therefore, we speculate that a small fraction of certain non-secreted RS1 mutants might reach the peripheral side of plasma membrane, although they are secretion incompetent. Upon reaching the plasma membrane, though not secreted, the mutant protein might perform varying degrees of cell adhesion and interaction function depending on the extent of structural damage conferred by the mutation. Although the interpretations arrived from *in vitro* and *in silico* experiments cannot be accounted in full for the *in vivo* cell machinery and effects, our data might provide an insight into the intricate pathophysiology of the disease. Thus, we restate the hypothesis that “the disease phenotype is not merely dependent on the secretory pattern of RS1, but, may be the precise localization of mutant RS1 in the cell and its overall structural conformation”. Hence, it would be a promising prospective study to examine the precise localization of the mutants as well as its ability to form a double octamer structure and likely to be functional.

### Conclusion

Though phenotype comparison between secreted and non-secreted group did not show any apparent difference, the secreted group presented with relatively severe disease indications than the non-secreted group. Moreover, there exists a wide intragroup variation. Besides, intrafamilial phenotype variability in XLRS still remains unresolved. However, these observations need to be tested in a large sample size to understand the true relationship between phenotype and genotype in XLRS patients. Altogether, this study emphasizes the fact that disease severity is not merely dependent on secretory profile of the mutations. It is proposed that the mutant protein’s ability to reach the exterior side of plasma membrane and the overall structural damage caused by the mutations might largely influence disease severity among patients showing the same secretion profile. With advanced methodologies, investigating the exact localization and structure of the mutant RS1 proteins would be feasible and thus the explicit molecular mechanism behind the pathogenesis of the disease could be unraveled.

## Supporting information

S1 Fig Representative illustration of the cloning of *RS1* mutant constructs by homologous recombination based Gibson assembly. (TIF)

S2 Fig Representative pedigrees of the affected XLRS families. (A) Family 3 showing patient 3 (III.2) and 4 (III.3). (B) Family 12 showing patient 14 (III.3) and 15 (III.4). (C) Family 17 showing patient 20 (III.1). (D) Family 8 showing patient 9 (III.2) and 10 (III.3). (E) Family 20 showing patient 23 (III.4) and 24 (II.4). (F) Family 19 showing patient 22 (III.4).
(TIF)

S3 Fig Pie chart showing the proportion of mutations in various domains of the *RS1* gene, identified in the Indian cohort. (TIF)

S4 Fig Graphical representation of ERG parameters between non-secreted and secreted group. (A) Scatter plot showing dark-adapted a-wave amplitude (μV) of both non-secreted and secreted group eyes. (B) Scatter plot showing dark-adapted b-wave amplitude (μV) of both non-secreted and secreted group eyes. The line in the graphs refers to the median values of normal individuals in μV.
(TIF)

S5 Fig Immunofluorescence localization of wild type (WT) and non-secreted mutant RS1 (Q129_144dup) in transfected COS7 cells (63X Magnification). (A) Co-staining of RS1 (green) along with plasma membrane (PM) marker calcium channel protein, CACNA1 (red). (B) Co-staining of RS1 (green) along with endoplasmic reticulum (ER) marker protein disulfide-isomerase, PDI (red). (C) Co-staining of RS1 (green) along with golgi apparatus (GA) marker golgin coiled-coil protein, golgin (red). Nucleus is stained with DAPI (blue).
(TIF)

S6 Fig Uncropped immunoblot image showing expression and secretion of WT and RS1 mutants. (TIF)

S1 Table Primer sequences used to create the *RS1* mutant constructs. (DOCX)

S2 Table cDNA sequence and molecular weight of the RS1 mutant proteins. (DOCX)

## References

[1] Peachey NS, Fishman GA, Derlacki DJ, Brigell MG. Psychophysical and electroretinographic findings in X-linked juvenile retinoschisis. Arch Ophthalmol (Chicago, Ill 1960). 1987;105: 513–516. 10.1001/archopht.1987.01060040083038 3566604

[2] The Retinoschisis Consortium. Functional implications of the spectrum of mutations found in 234 cases with X-linked juvenile retinoschisis (XLRS). 1998;7: 1185–1192. 10.1093/hmg/7.7.1185 9618178

[3] Sauer CG, Gehrig A, Warneke-Wittstock R, Marquardt A, Ewing CC, Gibson A, et al Positional cloning of the gene associated with X-linked juvenile retinoschisis. Nat Genet. 1997;17: 164–170. doi: 10.1038/ng1097-164 9326935 10.1038/ng1097-164

[4] Molday LL, Hicks D, Sauer CG, Weber BH, Molday RS. Expression of X-linked retinoschisis protein RS1 in photoreceptor and bipolar cells. Invest Ophthalmol Vis Sci. 2001;42: 816–825. 11222545

[5] Tolun G, Vijayasarathy C, Huang R, Zeng Y, Li Y, Steven AC. Paired octamer rings of retinoschisin suggest a junctional model for cell—cell adhesion in the retina. 2016;113: 5287–5292. 10.1073/pnas.1519048113 PMC4868477 27114531

[6] Wu WWH, Wong JP, Kast J, Molday RS. RS1 a discoidin domain-containing retinal cell adhesion protein associated with X-linked retinoschisis, exists as a novel disulfide-linked octamer. J Biol Chem. 2005;280: 10721–10730. doi: 10.1074/jbc.M413117200 15644328 10.1074/jbc.M413117200

[7] Molday RS. Focus on Molecules: Retinoschisin (RS1). Exp Eye Res. 2007;84: 227–228. doi: 10.1016/j.exer.2005.12.013 16600216 10.1016/j.exer.2005.12.013

[8] Li X, Ma X, Tao Y. Clinical features of X linked juvenile retinoschisis in Chinese families associated with novel mutations in the RS1 gene. Mol Vis. Emory University; 2007;13: 804–812. PMC2768756 17615541

[9] Wang N-K, Liu L, Chen H-M, Tsai S, Chang T-C, Tsai T-H, et al Clinical presentations of X-linked retinoschisis in Taiwanese patients confirmed with genetic sequencing. Mol Vis. Emory University; 2015;21: 487–501. PMC4415592 25999676

[10] George ND, Yates JR, Moore AT. Clinical features in affected males with X-linked retinoschisis. Arch Ophthalmol (Chicago, Ill 1960). 1996;114: 274–280. 10.1001/archopht.1996.01100130270007 8600886

[11] Eksandh LC, Ponjavic V, Ayyagari R, Bingham EL, Hiriyanna KT, Andréasson S, et al Phenotypic expression of juvenile X-linked retinoschisis in Swedish families with different mutations in the XLRS1 gene. Arch Ophthalmol (Chicago, Ill 1960). 2000;118: 1098–1104. 10.1001/archopht.118.8.1098 10922205

[12] Shinoda K, Ishida S, Oguchi Y, Mashima Y. Clinical characteristics of 14 japanese patients with X-linked juvenile retinoschisis associated with XLRS1 mutation. Ophthalmic Genet. 2000;21: 171–180. 11035549

[13] Pimenides D, George NDL, Yates JRW, Bradshaw K, Roberts SA, Moore AT, et al X-linked retinoschisis: clinical phenotype and RS1 genotype in 86 UK patients. J Med Genet. 2005;42: e35 doi: 10.1136/jmg.2004.029769 15937075 10.1136/jmg.2004.029769 PMC1736077

[14] Sergeev Y V., Caruso RC, Meltzer MR, Smaoui N, MacDonald IM, Sieving PA. Molecular modeling of retinoschisin with functional analysis of pathogenic mutations from human X-linked retinoschisis. Hum Mol Genet. 2010;19: 1302–1313. doi: 10.1093/hmg/ddq006 20061330 10.1093/hmg/ddq006 PMC2838538

[15] Wang T, Waters CT, Rothman AMK, Jakins TJ, Römisch K, Trump D. Intracellular retention of mutant retinoschisin is the pathological mechanism underlying X-linked retinoschisis. Hum Mol Genet. 2002;11: 3097–3105. 12417531 10.1093/hmg/11.24.3097

[16] Marmor MF, Fulton AB, Holder GE, Miyake Y, Brigell M, Bach M, et al ISCEV Standard for full-field clinical electroretinography (2008 update). Doc Ophthalmol. 2009;118: 69–77. doi: 10.1007/s10633-008-9155-4 19030905 10.1007/s10633-008-9155-4

[17] Sivakumaran TA, Igo RP, Kidd JM, Itsara A, Kopplin LJ, Chen W, et al A 32 kb critical region excluding Y402H in CFH mediates risk for age-related macular degeneration. PLoS One. 2011;6 (10): e25598 doi: 10.1371/journal.pone.0025598 22022419 10.1371/journal.pone.0025598 PMC3192039

[18] Kim SY, Ko HS, Yu YS, Hwang J, Lee JJ, Kim SY, et al Molecular genetic characteristics of X-linked retinoschisis in Koreans. 2009;15: 833–843. PMC2672147 19390641

[19] Wang T, Zhou A, Waters CT, O’Connor E, Read RJ, Trump D. Molecular pathology of X linked retinoschisis: mutations interfere with retinoschisin secretion and oligomerisation. Br J Ophthalmol. 2006;90: 81–86. doi: 10.1136/bjo.2005.078048 16361673 10.1136/bjo.2005.078048 PMC1856892

[20] Laskowski RA, MacArthur MW, Moss DS, Thornton JM, IUCr. PROCHECK: a program to check the stereochemical quality of protein structures. J Appl Crystallogr. International Union of Crystallography; 1993;26: 283–291.

[21] DeLano WL. The PyMOL Molecular Graphics System, Version 1.8. Schrödinger LLC. 2014; http://www.pymol.org.

[22] Molecular Operating Environment (MOE) 2013.08. Molecular Operating Environment (MOE), 2013.08; Chemical Computing Group Inc., 1010 Sherbooke St. West, Suite #910, Montreal, QC, Canada, H3A 2R7. Mol Oper Environ (MOE), 201308; Chem Comput Gr Inc, 1010 Sherbooke St West, Suite #910, Montr QC, Canada, H3A 2R7, 2013. 2016.

[23] Sudha D, Patric IRP, Ganapathy A, Agarwal S, Krishna S, Neriyanuri S, et al Genetic studies in a patient with X-linked retinoschisis coexisting with developmental delay and sensorineural hearing loss. Ophthalmic Genet. Taylor & Francis; 2017;38: 260–266. 10.1080/13816810.2016.1214972 28574807

[24] Neriyanuri S, Dhandayuthapani S, Arunachalam J, Raman R. Phenotypic characterization of X-linked retinoschisis: Clinical, electroretinography, and optical coherence tomography variables. Indian J Ophthalmol. 2016;64: 513–517. doi: 10.4103/0301-4738.190140 27609164 10.4103/0301-4738.190140 PMC5026077

[25] Wu WWH, Molday RS. Defective discoidin domain structure, subunit assembly, and endoplasmic reticulum processing of retinoschisin are primary mechanisms responsible for X-linked retinoschisis. J Biol Chem. 2003;278: 28139–28146. doi: 10.1074/jbc.M302464200 12746437 10.1074/jbc.M302464200

[26] Vijayasarathy C, Sui R, Zeng Y, Yang G, Xu F, Caruso RC, et al Molecular mechanisms leading to null-protein product from retinoschisin (RS1) signal-sequence mutants in X-Linked Retinoschisis (XLRS) disease. Hum Mutat. 2010;31: 1251–1260. doi: 10.1002/humu.21350 20809529 10.1002/humu.21350 PMC2991635

[27] Ramsay EP, Collins RF, Owens TW, Siebert CA, Jones RPO, Wang T, et al Structural analysis of X-linked retinoschisis mutations reveals distinct classes which differentially effect retinoschisin function. 2016;25: 5311–5320. 10.1093/hmg/ddw345 PMC5418834 27798099

[28] Sudha D, Kohansal-Nodehi M, Kovuri P, Manda SS, Neriyanuri S, Gopal L, et al Proteomic profiling of human intraschisis cavity fluid. Clin Proteomics. 2017;14: 13 doi: 10.1186/s12014-017-9148-y 28450823 10.1186/s12014-017-9148-y PMC5404285

[29] Vincent A, Robson AG, Neveu MM, Wright GA, Moore AT, Webster AR, et al A phenotype-genotype correlation study of X-linked retinoschisis. Ophthalmology. 2013;120: 1454–1464. doi: 10.1016/j.ophtha.2012.12.008 23453514 10.1016/j.ophtha.2012.12.008

[30] Noble KG, Margolis S, Carr RE. The golden tapetal sheen reflex in retinal disease. Am J Ophthalmol. 1989;107: 211–217. 2923149 10.1016/0002-9394(89)90302-4

[31] de Jong PT, Zrenner E, van Meel GJ, Keunen JE, van Norren D. Mizuo phenomenon in X-linked retinoschisis. Pathogenesis of the Mizuo phenomenon. Arch Ophthalmol (Chicago, Ill 1960). 1991;109: 1104–1108. 10.1001/archopht.1991.01080080064029 1867553

[32] Iannaccone A, Mura M, Dyka FM, Laura M, Yashar BM, Ayyagari R, et al An unusual X-linked retinoschisis phenotype and biochemical characterization of the W112C RS1 mutation. 2006;46: 3845–3852. 10.1016/j.visres.2006.06.011 16884758

[33] Vijayasarathy C, Ziccardi L, Zeng Y, Smaoui N, Caruso RC, Sieving PA. Null retinoschisin-protein expression from an RS1 c354del1-ins18 mutation causing progressive and severe XLRS in a cross-sectional family study. Investig Ophthalmol Vis Sci. 2009;50: 5375–5383. 19474399 10.1167/iovs.09-3839 PMC2784021

[34] Steiner-Champliaud M-F, Sahel J, Hicks D. Retinoschisin forms a multi-molecular complex with extracellular matrix and cytoplasmic proteins: interactions with beta2 laminin and alphaB-crystallin. Mol Vis. 2006;12: 892–901. 16917482

[35] Shi L, Jian K, Ko ML, Trump D, Ko GYP. Retinoschisin, a new binding partner for L-type voltage-gated calcium channels in the retina. J Biol Chem. 2009;284: 3966–3975. doi: 10.1074/jbc.M806333200 19074145 10.1074/jbc.M806333200 PMC2635055

[36] Johnson BA, Cole BS, Geisert EE, Ikeda S, Ikeda A. Tyrosinase is the modifier of retinoschisis in mice. Genetics. 2010;186: 1337–1344. doi: 10.1534/genetics.110.120840 20876567 10.1534/genetics.110.120840 PMC2998315

[37] Vijayasarathy C, Takada Y, Zeng Y, Bush RA, Sieving PA. Retinoschisin is a peripheral membrane protein with affinity for anionic phospholipids and affected by divalent cations. Invest Ophthalmol Vis Sci. 2007;48: 991–1000. doi: 10.1167/iovs.06-0915 17325137 10.1167/iovs.06-0915

