# Supplementary figures and images for: Understanding variable disease severity in X-linked retinoschisis: Does RS1 secretory mechanism determine disease severity?
Source: PLoS One. 2018 May 31;13(5):e0198086. doi: 10.1371/journal.pone.0198086 (PMC5978886; doi:10.1371/journal.pone.0198086)

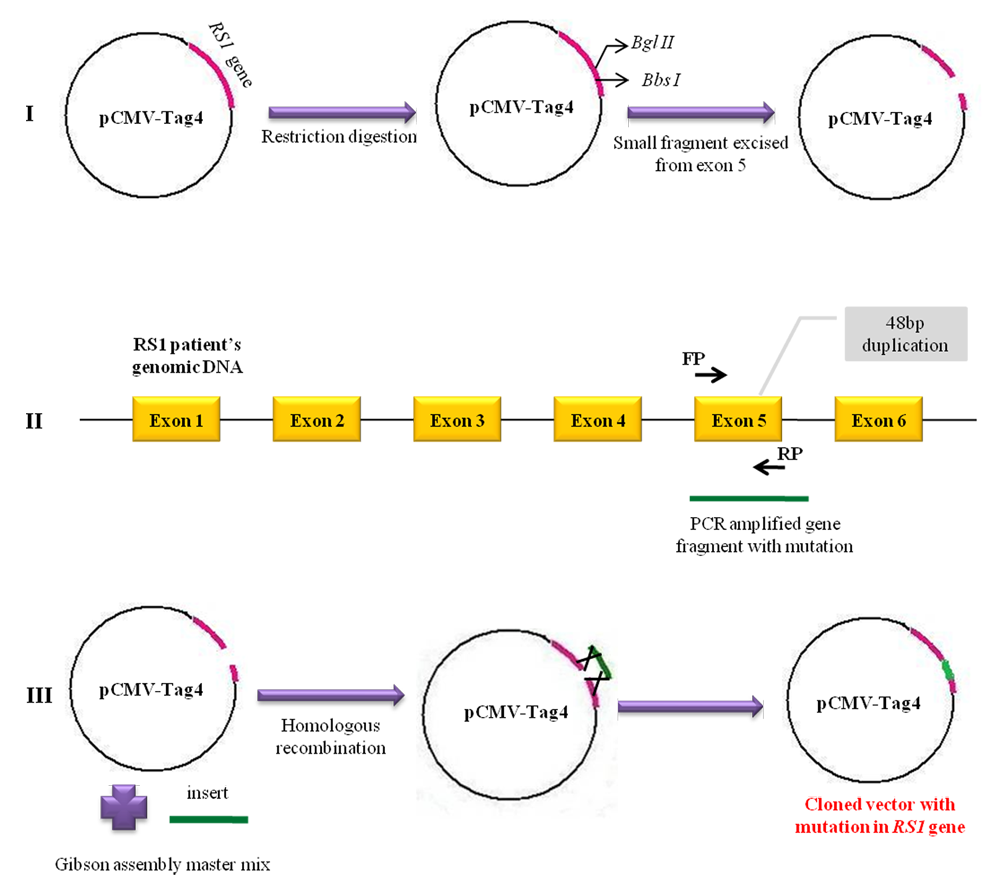

Supplement: S1 Fig — (TIF) [file pone.0198086.s001.tif]

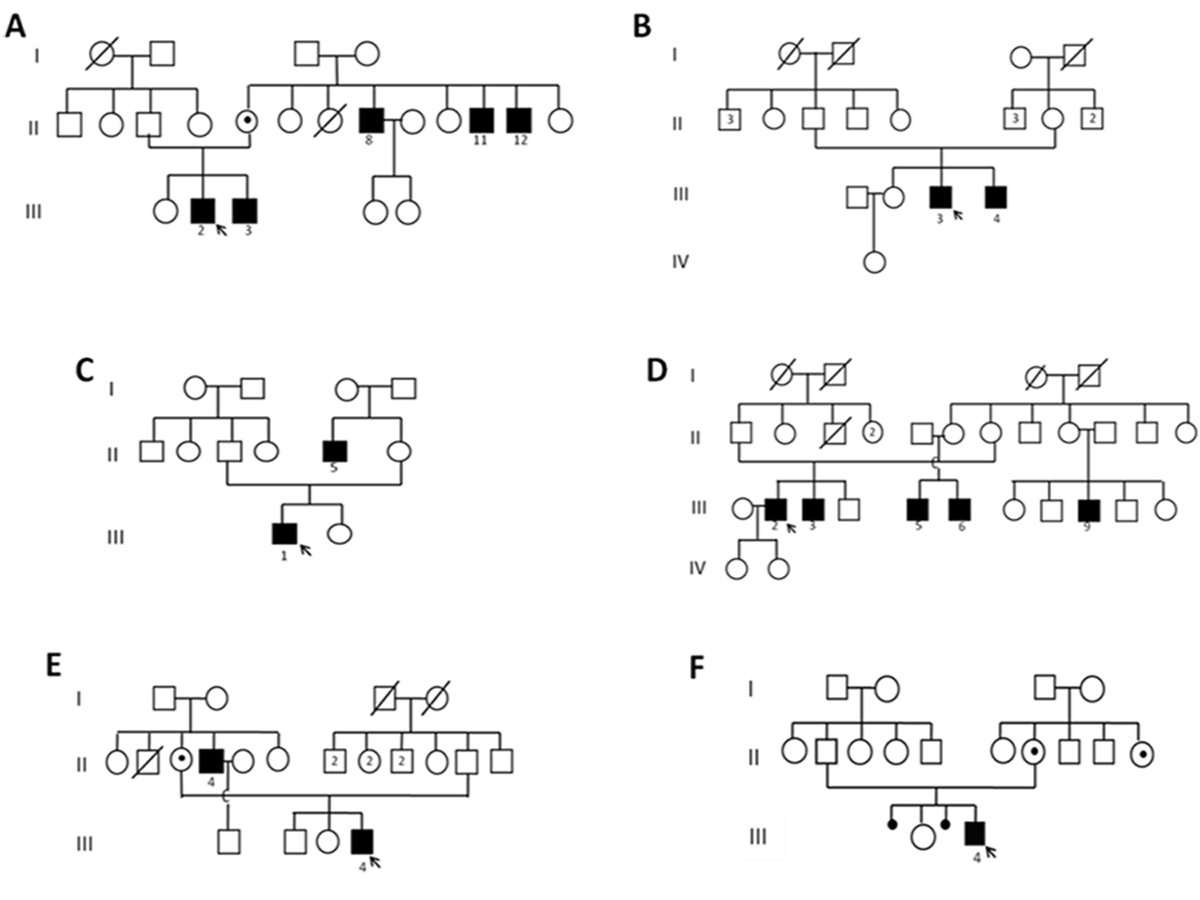

Supplement: S2 Fig — (A) Family 3 showing patient 3 (III.2) and 4 (III.3). (B) Family 12 showing patient 14 (III.3) and 15 (III.4). (C) Family 17 showing patient 20 (III.1). (D) Family 8 showing patient 9 (III.2) and 10 (III.3). (E) Family 20 showing patient 23 (III.4) and 24 (II.4). (F) Family 19 showing patient 22 (III.4). (TIF) [file pone.0198086.s002.tif]

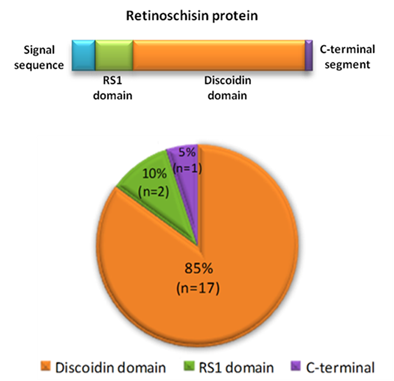

Supplement: S3 Fig — (TIF) [file pone.0198086.s003.tif]

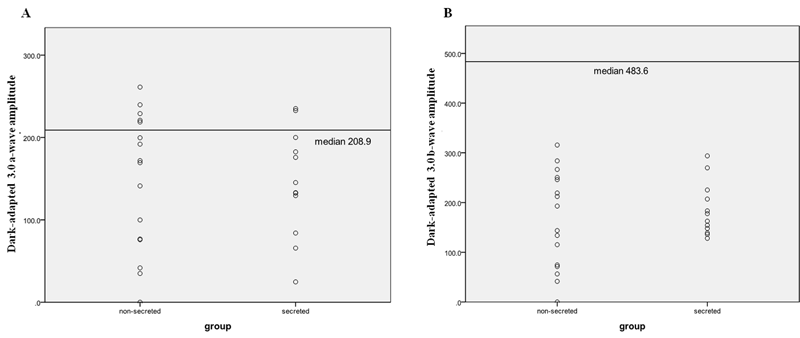

Supplement: S4 Fig — (A) Scatter plot showing dark-adapted a-wave amplitude (μV) of both non-secreted and secreted group eyes. (B) Scatter plot showing dark-adapted b-wave amplitude (μV) of both non-secreted and secreted group eyes. The line in the graphs refers to the median values of normal individuals in μV. (TIF) [file pone.0198086.s004.tif]

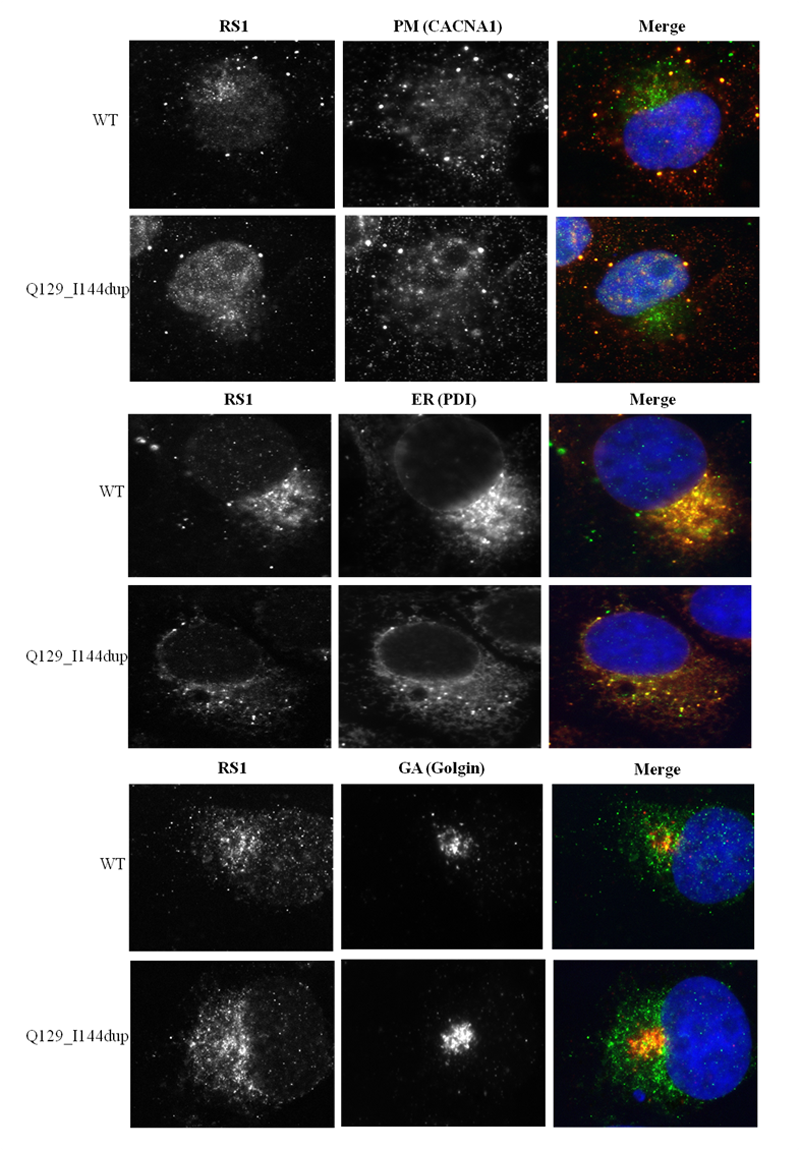

Supplement: S5 Fig — (A) Co-staining of RS1 (green) along with plasma membrane (PM) marker calcium channel protein, CACNA1 (red). (B) Co-staining of RS1 (green) along with endoplasmic reticulum (ER) marker protein disulfide-isomerase, PDI (red). (C) Co-staining of RS1 (green) along with golgi apparatus (GA) marker golgin coiled-coil protein, golgin (red). Nucleus is stained with DAPI (blue). (TIF) [file pone.0198086.s005.tif]

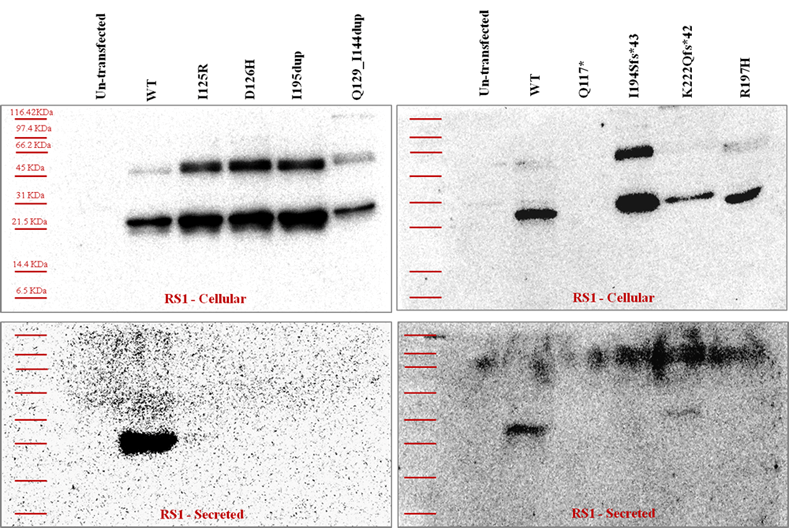

Supplement: S6 Fig — (TIF) [file pone.0198086.s006.tif]
