# Supplementary material for: Understanding variable disease severity in X-linked retinoschisis: Does RS1 secretory mechanism determine disease severity?
Source: PLoS One. 2018 May 31;13(5):e0198086. doi: 10.1371/journal.pone.0198086 (PMC5978886; doi:10.1371/journal.pone.0198086)
Supplement: S1 Table — (DOCX) [file pone.0198086.s007.docx]

| **S1 Table. Primer sequences used to create the *RS1* mutant constructs** | | | | |
| --- | --- | --- | --- | --- |
| **MUTATION** | **PRIMER SEQUENCE (5'- 3')** | **RE ENZYMES** | **ANNEALING TEMP (°C)** | **METHOD** |
| D126H | FP:TAGCCAGTGGTTACAGATACATCTGAAGGAGATCAAAG RP:CTTTGATCTCCTTCAGATGTATCTGTAACCACTGGCTA | *DpnI* | 60 | Quickchange kit |
| I195dup | FP:CTGCGGCCCCCCATCATCATCTCCCGC RP:GCGGGAGATGATGATGGGGGGCCGCAG | *DpnI* | 75 | Quickchange kit |
| I125R | FP:CTAGCGATTACGCCAAGCTC RP:CCCCTGGGTGAGGATCCCTGAAATCACTTTGATCTCCTTCAGATCTCTCTGTAACCACTGGCTACTGTCC T | *NotI;BglII* | 60 | Gibson assembly kit |
| Q129_I144dup | FP:GTGTGCCTGGCTCTCCAAGTTCCA RP:AGGTTCTGAACCGTGGAGGTGCGGTCCGAGTTGCCATAGAAGAC CCGGTTGTTTCCAGTCTGGTCCTT | *BglII;BbsI* | 60 | Gibson assembly |
| K222Qfs*42 | FP:GTCTTCTATGGCAACTCGGACCGCA RP:CTACTTATCGTCGTCATCCTTGTAATCCAATTGCTTTGCGAAATATAGCCCT | *BmgBI;Xho I* | 60 | Gibson assembly kit |
| I194Sfs*43 | FP_1_:GTGTGCCTGGCTCTCCAAGTTCCA RP_1_:TGCGGTCCGAGTTGCCATAGAAGACCCGGTTGTTTCCAGTCTGGTCCTTG FP_2_:GTCTTCTATGGCAACTCGGACCGCA RP_2_:CTACTTATCGTCGTCATCCTTGTAATCCCCCCTGGCAGGCGCCGAGCTGAGG | *BbsI;XhoI* | 60;54 | Gibson assembly kit |
| R197H | FP:CTAGCGATTACGCCAAGCTC RP:AGCAGCTCCATCCGGATGGCAATGCGGACGTGCCAGCCCAGCGGGATGAGGCGGATGAAGTGGGAGATGATGGGGGGCCGC | *NotI;BmgBI* | 60 | Gibson assembly kit |
| RE – Restriction enzyme; Temp - Temperature | | | | |
