# Supplementary material for: Understanding variable disease severity in X-linked retinoschisis: Does RS1 secretory mechanism determine disease severity?
Source: PLoS One. 2018 May 31;13(5):e0198086. doi: 10.1371/journal.pone.0198086 (PMC5978886; doi:10.1371/journal.pone.0198086)
Supplement: S2 Table — (DOCX) [file pone.0198086.s008.docx]

| **S2 Table. cDNA Sequence and molecular weight of the RS1 mutant proteins** | | |
| --- | --- | --- |
| **RS1** | **cDNA SEQUENCE (5'- 3')** | **CALCULATED MOLECULAR WEIGHT OF PROTEIN PRODUCTS (KDa)** |
| WT | ATGTCACGCAAGATAGAAGGCTTTTTGTTATTACTTCTCTTTGGCTATGAAGCCACATTGGGATTATCGTCTACCGAGGATGAAGGCGAGGACCCCTGGTACCAAAAAGCATGCAAGTGCGATTGCCAAGGAGGACCCAATGCTCTGTGGTCTGCAGGTGCCACCTCCTTGGACTGTATACCAGAATGCCCATATCACAAGCCTCTGGGTTTCGAGTCAGGGGAGGTCACACCGGACCAGATCACCTGCTCTAACCCGGAGCAGTATGTGGGCTGGTATTCTTCGTGGACTGCAAACAAGGCCCGGCTCAACAGTCAAGGCTTTGGGTGTGCCTGGCTCTCCAAGTTCCAGGACAGTAGCCAGTGGTTACAGATAGATCTGAAGGAGATCAAAGTGATTTCAGGGATCCTCACCCAGGGGCGCTGTGACATCGATGAGTGGATGACCAAGTACAGCGTGCAGTACAGGACCGATGAGCGCCTGAACTGGATTTACTACAAGGACCAGACTGGAAACAACCGGGTCTTCTATGGCAACTCGGACCGCACCTCCACGGTTCAGAACCTGCTGCGGCCCCCCATCATCTCCCGCTTCATCCGCCTCATCCCGCTGGGCTGGCACGTCCGCATTGCCATCCGGATGGAGCTGCTGGAGTGCGTCAGCAAGTGTGCCCTCGAGGATTACAAGGATGACGACGATAAGTAG | 24 |
| I125R | ATGTCACGCAAGATAGAAGGCTTTTTGTTATTACTTCTCTTTGGCTATGAAGCCACATTGGGATTATCGTCTACCGAGGATGAAGGCGAGGACCCCTGGTACCAAAAAGCATGCAAGTGCGATTGCCAAGGAGGACCCAATGCTCTGTGGTCTGCAGGTGCCACCTCCTTGGACTGTATACCAGAATGCCCATATCACAAGCCTCTGGGTTTCGAGTCAGGGGAGGTCACACCGGACCAGATCACCTGCTCTAACCCGGAGCAGTATGTGGGCTGGTATTCTTCGTGGACTGCAAACAAGGCCCGGCTCAACAGTCAAGGCTTTGGGTGTGCCTGGCTCTCCAAGTTCCAGGACAGTAGCCAGTGGTTACAGAGAGATCTGAAGGAGATCAAAGTGATTTCAGGGATCCTCACCCAGGGGCGCTGTGACATCGATGAGTGGATGACCAAGTACAGCGTGCAGTACAGGACCGATGAGCGCCTGAACTGGATTTACTACAAGGACCAGACTGGAAACAACCGGGTCTTCTATGGCAACTCGGACCGCACCTCCACGGTTCAGAACCTGCTGCGGCCCCCCATCATCTCCCGCTTCATCCGCCTCATCCCGCTGGGCTGGCACGTCCGCATTGCCATCCGGATGGAGCTGCTGGAGTGCGTCAGCAAGTGTGCCCTCGAGGATTACAAGGATGACGACGATAAGTAG | 24 |
| D126H | ATGTCACGCAAGATAGAAGGCTTTTTGTTATTACTTCTCTTTGGCTATGAAGCCACATTGGGATTATCGTCTACCGAGGATGAAGGCGAGGACCCCTGGTACCAAAAAGCATGCAAGTGCGATTGCCAAGGAGGACCCAATGCTCTGTGGTCTGCAGGTGCCACCTCCTTGGACTGTATACCAGAATGCCCATATCACAAGCCTCTGGGTTTCGAGTCAGGGGAGGTCACACCGGACCAGATCACCTGCTCTAACCCGGAGCAGTATGTGGGCTGGTATTCTTCGTGGACTGCAAACAAGGCCCGGCTCAACAGTCAAGGCTTTGGGTGTGCCTGGCTCTCCAAGTTCCAGGACAGTAGCCAGTGGTTACAGATACATCTGAAGGAGATCAAAGTGATTTCAGGGATCCTCACCCAGGGGCGCTGTGACATCGATGAGTGGATGACCAAGTACAGCGTGCAGTACAGGACCGATGAGCGCCTGAACTGGATTTACTACAAGGACCAGACTGGAAACAACCGGGTCTTCTATGGCAACTCGGACCGCACCTCCACGGTTCAGAACCTGCTGCGGCCCCCCATCATCTCCCGCTTCATCCGCCTCATCCCGCTGGGCTGGCACGTCCGCATTGCCATCCGGATGGAGCTGCTGGAGTGCGTCAGCAAGTGTGCCCTCGAGGATTACAAGGATGACGACGATAAGTAG | 24 |
| I195dup | ATGTCACGCAAGATAGAAGGCTTTTTGTTATTACTTCTCTTTGGCTATGAAGCCACATTGGGATTATCGTCTACCGAGGATGAAGGCGAGGACCCCTGGTACCAAAAAGCATGCAAGTGCGATTGCCAAGGAGGACCCAATGCTCTGTGGTCTGCAGGTGCCACCTCCTTGGACTGTATACCAGAATGCCCATATCACAAGCCTCTGGGTTTCGAGTCAGGGGAGGTCACACCGGACCAGATCACCTGCTCTAACCCGGAGCAGTATGTGGGCTGGTATTCTTCGTGGACTGCAAACAAGGCCCGGCTCAACAGTCAAGGCTTTGGGTGTGCCTGGCTCTCCAAGTTCCAGGACAGTAGCCAGTGGTTACAGATAGATCTGAAGGAGATCAAAGTGATTTCAGGGATCCTCACCCAGGGGCGCTGTGACATCGATGAGTGGATGACCAAGTACAGCGTGCAGTACAGGACCGATGAGCGCCTGAACTGGATTTACTACAAGGACCAGACTGGAAACAACCGGGTCTTCTATGGCAACTCGGACCGCACCTCCACGGTTCAGAACCTGCTGCGGCCCCCCATCATCATCTCCCGCTTCATCCGCCTCATCCCGCTGGGCTGGCACGTCCGCATTGCCATCCGGATGGAGCTGCTGGAGTGCGTCAGCAAGTGTGCCCTCGAGGATTACAAGGATGACGACGATAAGTAG | 24 |
| Q129_I144dup | ATGTCACGCAAGATAGAAGGCTTTTTGTTATTACTTCTCTTTGGCTATGAAGCCACATTGGGATTATCGTCTACCGAGGATGAAGGCGAGGACCCCTGGTACCAAAAAGCATGCAAGTGCGATTGCCAAGGAGGACCCAATGCTCTGTGGTCTGCAGGTGCCACCTCCTTGGACTGTATACCAGAATGCCCATATCACAAGCCTCTGGGTTTCGAGTCAGGGGAGGTCACACCGGACCAGATCACCTGCTCTAACCCGGAGCAGTATGTGGGCTGGTATTCTTCGTGGACTGCAAACAAGGCCCGGCTCAACAGTCAAGGCTTTGGGTGTGCCTGGCTCTCCAAGTTCCAGGACAGTAGCCAGTGGTTACAGATAGATCTGAAGGAGATCAAAGTGATTTCAGGGATCCTCACCCAGGGGCGCTGTGACATCGAGATCAAAGTGATTTCAGGGATCCTCACCCAGGGGCGCTGTGACATCGATGAGTGGATGACCAAGTACAGCGTGCAGTACAGGACCGATGAGCGCCTGAACTGGATTTACTACAAGGACCAGACTGGAAACAACCGGGTCTTCTATGGCAACTCGGACCGCACCTCCACGGTTCAGAACCTGCTGCGGCCCCCCATCATCTCCCGCTTCATCCGCCTCATCCCGCTGGGCTGGCACGTCCGCATTGCCATCCGGATGGAGCTGCTGGAGTGCGTCAGCAAGTGTGCCCTCGAGGATTACAAGGATGACGACGATAAGTAG | 26 |
| Q117* | ATGTCACGCAAGATAGAAGGCTTTTTGTTATTACTTCTCTTTGGCTATGAAGCCACATTGGGATTATCGTCTACCGAGGATGAAGGCGAGGACCCCTGGTACCAAAAAGCATGCAAGTGCGATTGCCAAGGAGGACCCAATGCTCTGTGGTCTGCAGGTGCCACCTCCTTGGACTGTATACCAGAATGCCCATATCACAAGCCTCTGGGTTTCGAGTCAGGGGAGGTCACACCGGACCAGATCACCTGCTCTAACCCGGAGCAGTATGTGGGCTGGTATTCTTCGTGGACTGCAAACAAGGCCCGGCTCAACAGTCAAGGCTTTGGGTGTGCCTGGCTCTCCAAGTTCGATTACAAGGATGACGACGATAAGTAG | 12 |
| I194Sfs*43 | ATGTCACGCAAGATAGAAGGCTTTTTGTTATTACTTCTCTTTGGCTATGAAGCCACATTGGGATTATCGTCTACCGAGGATGAAGGCGAGGACCCCTGGTACCAAAAAGCATGCAAGTGCGATTGCCAAGGAGGACCCAATGCTCTGTGGTCTGCAGGTGCCACCTCCTTGGACTGTATACCAGAATGCCCATATCACAAGCCTCTGGGTTTCGAGTCAGGGGAGGTCACACCGGACCAGATCACCTGCTCTAACCCGGAGCAGTATGTGGGCTGGTATTCTTCGTGGACTGCAAACAAGGCCCGGCTCAACAGTCAAGGCTTTGGGTGTGCCTGGCTCTCCAAGTTCCAGGACAGTAGCCAGTGGTTACAGATAGATCTGAAGGAGATCAAAGTGATTTCAGGGATCCTCACCCAGGGGCGCTGTGACATCGATGAGTGGATGACCAAGTACAGCGTGCAGTACAGGACCGATGAGCGCCTGAACTGGATTTACTACAAGGACCAGACTGGAAACAACCGGGTCTTCTATGGCAACTCGGACCGCACCTCCACGGTTCAGAACCTGCTGCGGCCCCCATCATCTCCCGCTTCATCCGCCTCATCCCGCTGGGCTGGCACGTCCGCATTGCCATCCGGATGGAGCTGCTGGAGTGCGTCAGCAAGTGTGCCTGATGCCTGCCTCAGCTCGGCGCCTGCCAGGGGGGATT ACAAGGATGACGACGATAAGTAG | 25 |
| K222Qfs*42 | ATGTCACGCAAGATAGAAGGCTTTTTGTTATTACTTCTCTTTGGCTATGAAGCCACATTGGGATTATCGTCTACCGAGGATGAAGGCGAGGACCCCTGGTACCAAAAAGCATGCAAGTGCGATTGCCAAGGAGGACCCAATGCTCTGTGGTCTGCAGGTGCCACCTCCTTGGACTGTATACCAGAATGCCCATATCACAAGCCTCTGGGTTTCGAGTCAGGGGAGGTCACACCGGACCAGATCACCTGCTCTAACCCGGAGCAGTATGTGGGCTGGTATTCTTCGTGGACTGCAAACAAGGCCCGGCTCAACAGTCAAGGCTTTGGGTGTGCCTGGCTCTCCAAGTTCCAGGACAGTAGCCAGTGGTTACAGATAGATCTGAAGGAGATCAAAGTGATTTCAGGGATCCTCACCCAGGGGCGCTGTGACATCGATGAGTGGATGACCAAGTACAGCGTGCAGTACAGGACCGATGAGCGCCTGAACTGGATTTACTACAAGGACCAGACTGGAAACAACCGGGTCTTCTATGGCAACTCGGACCGCACCTCCACGGTTCAGAACCTGCTGCGGCCCCCCATCATCTCCCGCTTCATCCGCCTCATCCCGCTGGGCTGGCACGTCCGCATTGCCATCCGGATGGAGCTGCTGGAGTGCGTCAGCCAAGTGTGCCTGATGCCTGCCTCAGCTCGGCGCCTGCCAGGGGGTGACTGGCACAGAGCGGGCCGTAGGGGACCCCCTCACACACCACCGAGATGGACAGGGCTATATTTCGCAAAGCAATTGGATTACAAGGATGACGACGATAAGTAG | 28 |
| R197H | ATGTCACGCAAGATAGAAGGCTTTTTGTTATTACTTCTCTTTGGCTATGAAGCCACATTGGGATTATCGTCTACCGAGGATGAAGGCGAGGACCCCTGGTACCAAAAAGCATGCAAGTGCGATTGCCAAGGAGGACCCAATGCTCTGTGGTCTGCAGGTGCCACCTCCTTGGACTGTATACCAGAATGCCCATATCACAAGCCTCTGGGTTTCGAGTCAGGGGAGGTCACACCGGACCAGATCACCTGCTCTAACCCGGAGCAGTATGTGGGCTGGTATTCTTCGTGGACTGCAAACAAGGCCCGGCTCAACAGTCAAGGCTTTGGGTGTGCCTGGCTCTCCAAGTTCCAGGACAGTAGCCAGTGGTTACAGATAGATCTGAAGGAGATCAAAGTGATTTCAGGGATCCTCACCCAGGGGCGCTGTGACATCGATGAGTGGATGACCAAGTACAGCGTGCAGTACAGGACCGATGAGCGCCTGAACTGGATTTACTACAAGGACCAGACTGGAAACAACCGGGTCTTCTATGGCAACTCGGACCGCACCTCCACGGTTCAGAACCTGCTGCGGCCCCCCATCATCTCCCACTTCATCCGCCTCATCCCGCTGGGCTGGCACGTCCGCATTGCCATCCGGATGGAGCTGCTGGAGTGCGTCAGCAAGTGTGCCCTCGAGGATTACAAGGATGCGACGATAAGTAG | 24 |
